# Supplementary material for: Average miniature post-synaptic potential size is inversely proportional to membrane capacitance across neocortical pyramidal neurons of different sizes
Source: Front Cell Neurosci. 2025 Jun 18;19:1590157. doi: 10.3389/fncel.2025.1590157 (PMC12213790; doi:10.3389/fncel.2025.1590157)
Supplement: Supplementary file 1 [file Data_Sheet_1.docx]

**Supplementary Figures**


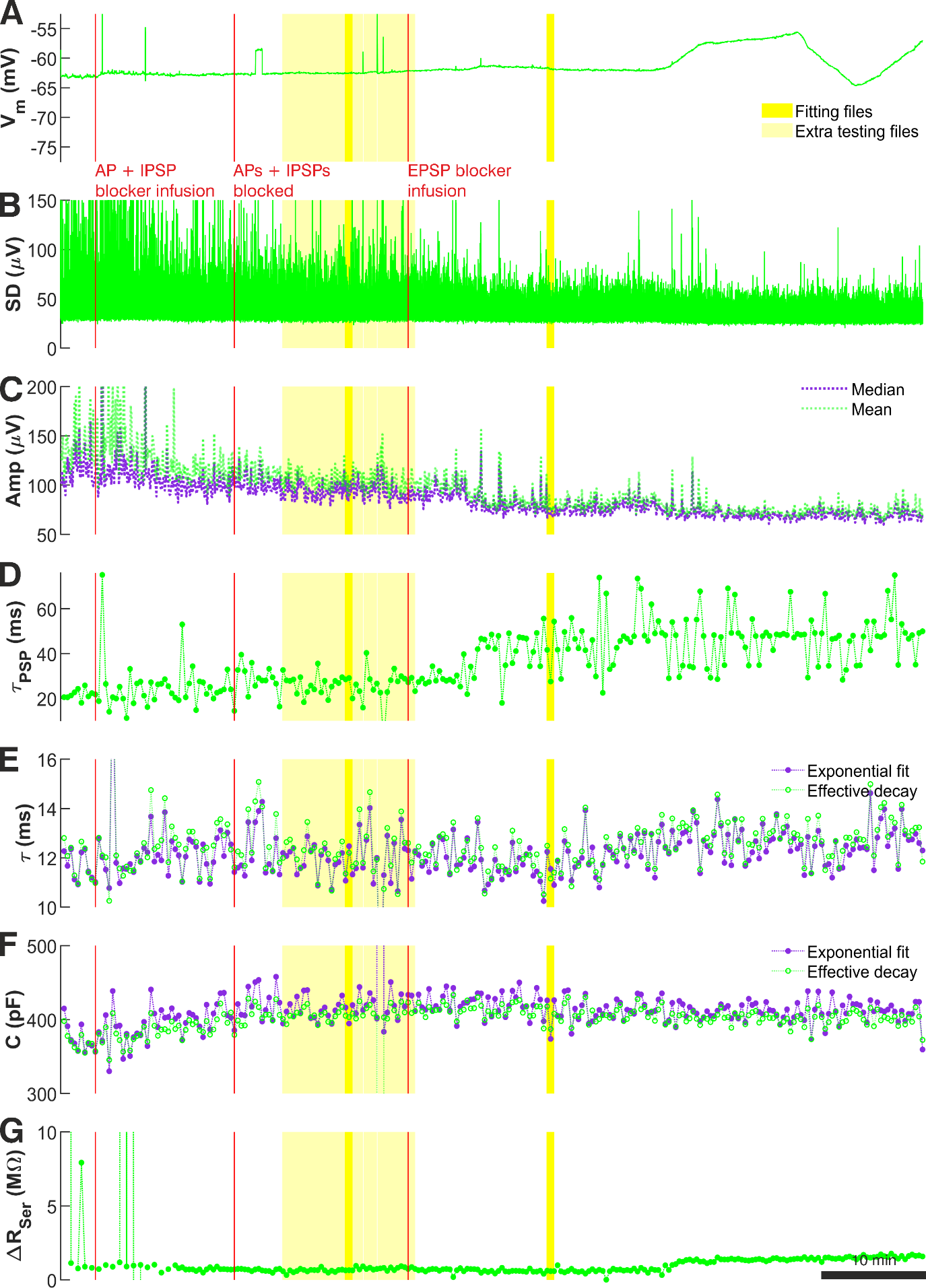
**Supplementary Figure 1:** Cell p106b (layer 5). Recording quality measures used to select ’noise with minis’ and ’noise-alone’ sweeps.

(A) Baseline V_m_ across entire recording (excluding stimulation pulse periods); duration 4960 s. Red lines and shaded yellow regions and Panels B-G as for Figure 2.


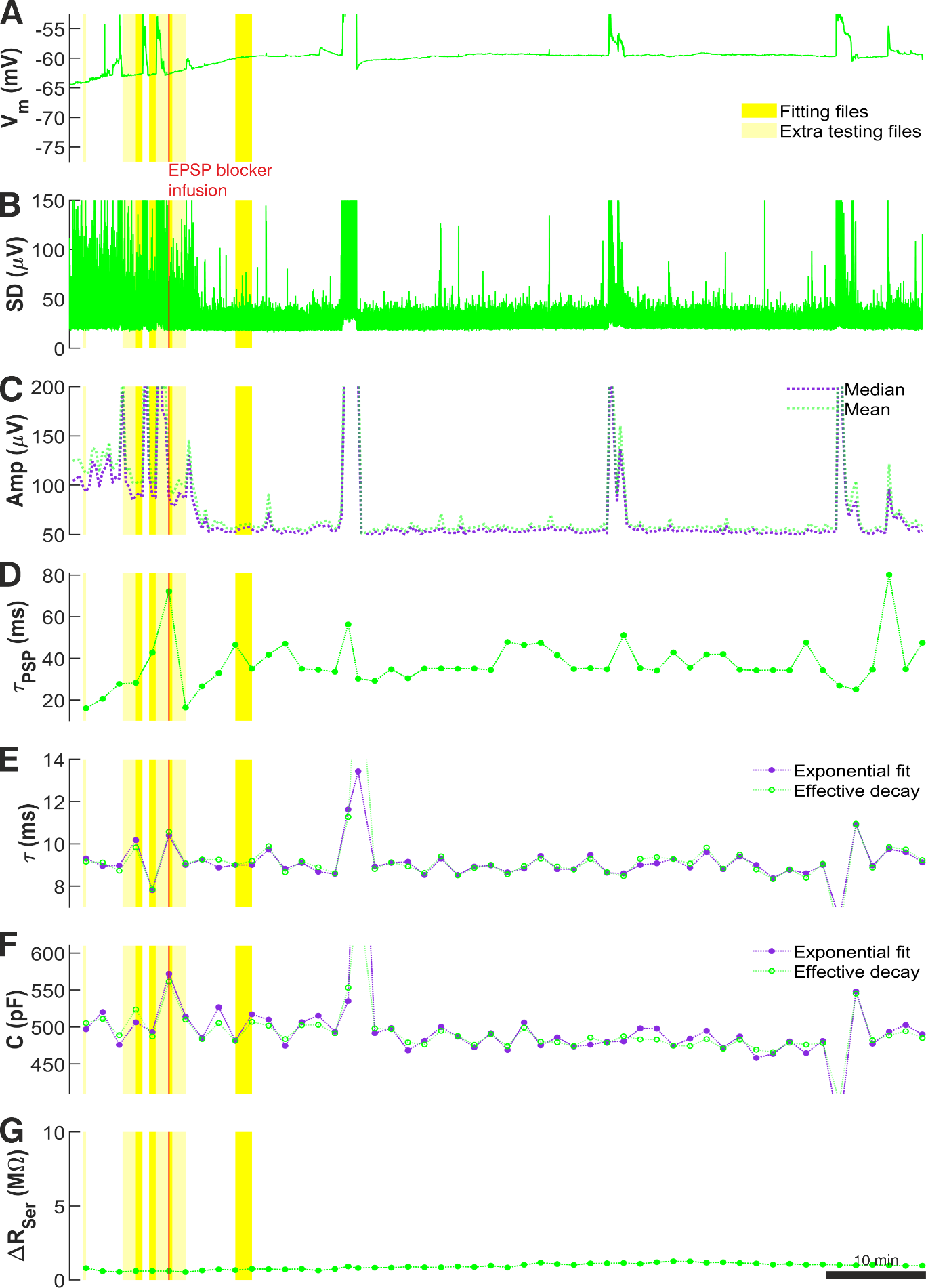
**Supplementary Figure 2:** Cell p108a (layer 5). Recording quality measures used to select ’noise with minis’ and ’noise-alone’ sweeps.

Panels A-G as for Figure 1 except for data being averaged over 100-second-long windows (5 recording sweeps of 20 s each) in panels D-G. The figure shows only part of the recording following the blockade of APs with only a single red vertical time marker visible indicating the time when pharmacological EPSP blockers were infused. The total duration of the recording shown in the figure was 5140 s. Some episodes with transient recording instabilities (noise glitches) were removed from the ‘noise with minis’ epoch (white gaps).


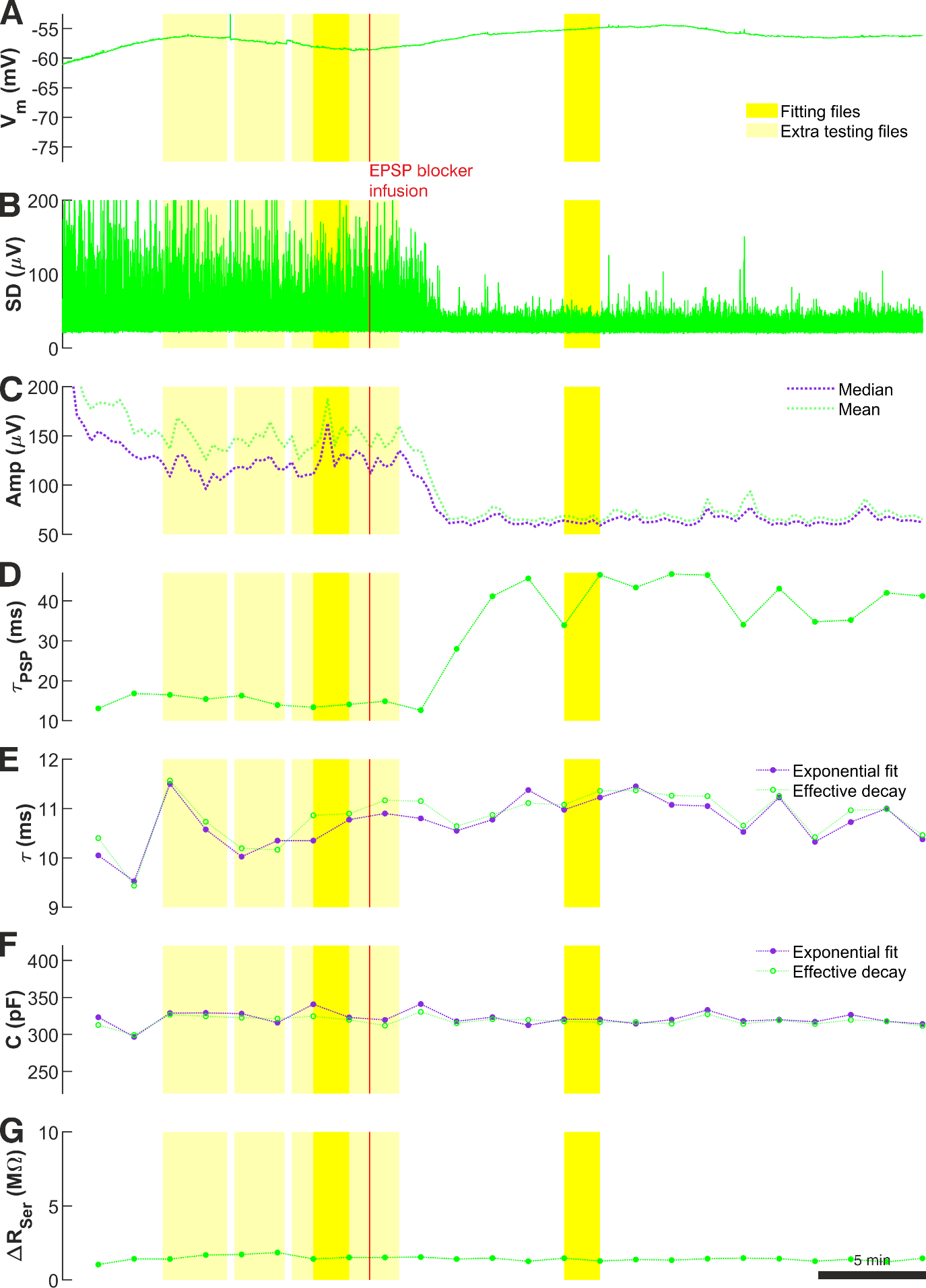
**Supplementary Figure 3:** Cell p108b (layer 5). Recording quality measures used to select ’noise with minis’ and ’noise-alone’ sweeps.

Panels A-G as for Figure 2 and Supplementary Figure 2. Data were averaged over 100-second-long windows (5 recording sweeps of 20 s each) in panels D-G; duration 2400 s.


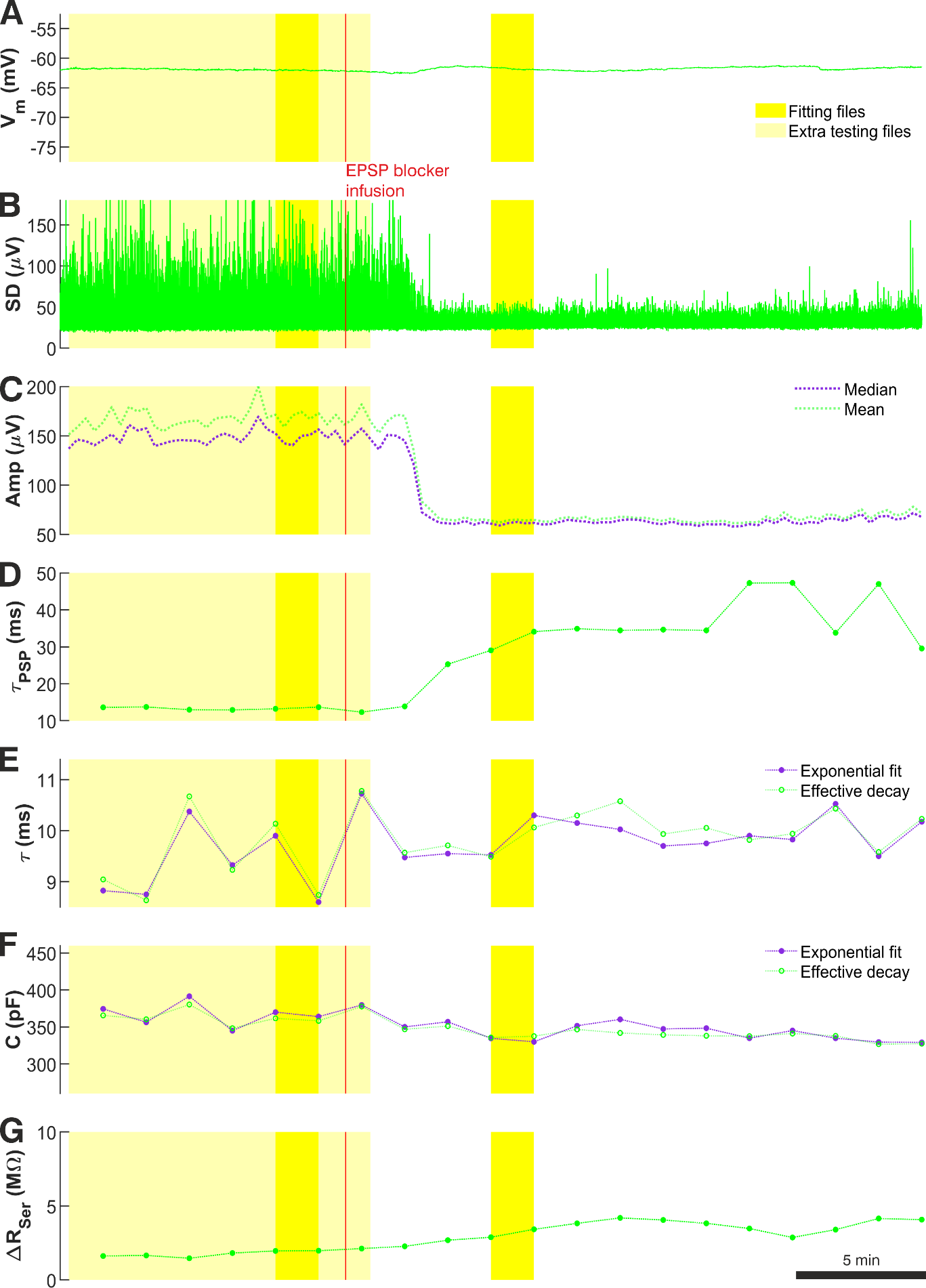
**Supplementary Figure 4:** Cell p108c (layer 5). Recording quality measures used to select ’noise with minis’ and ’noise-alone’ sweeps.

Panels A-G as for Figure 2 and Supplementary Figure 2. Data were averaged over 100-second-long windows (5 recording sweeps of 20 s each) in panels D-G; duration 2000 s.


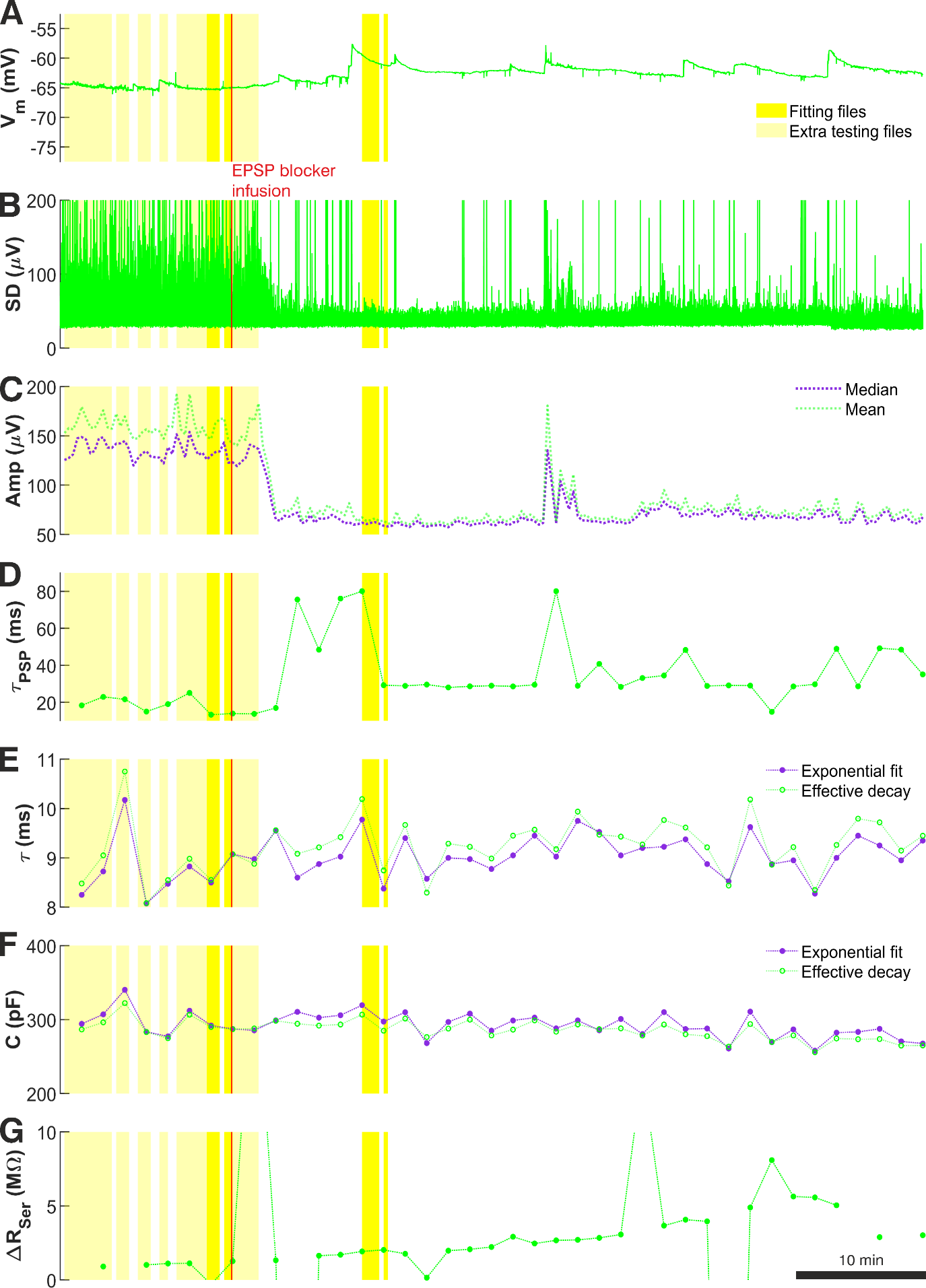
**Supplementary Figure 5:** Cell p120b (layer 2/3). Recording quality measures used to select ’noise with minis’ and ’noise-alone’ sweeps.

Panels A-G as for Figure 2 and Supplementary Figure 2. Data were averaged over 100-second-long windows (5 recording sweeps of 20 s each) in panels D-G; duration 4000 s. Some transient ‘glitchy’ extra noisy periods (due to transient recording instability) were manually excluded from the ‘noise with minis’ and ‘noise-alone’ epochs (white gaps).


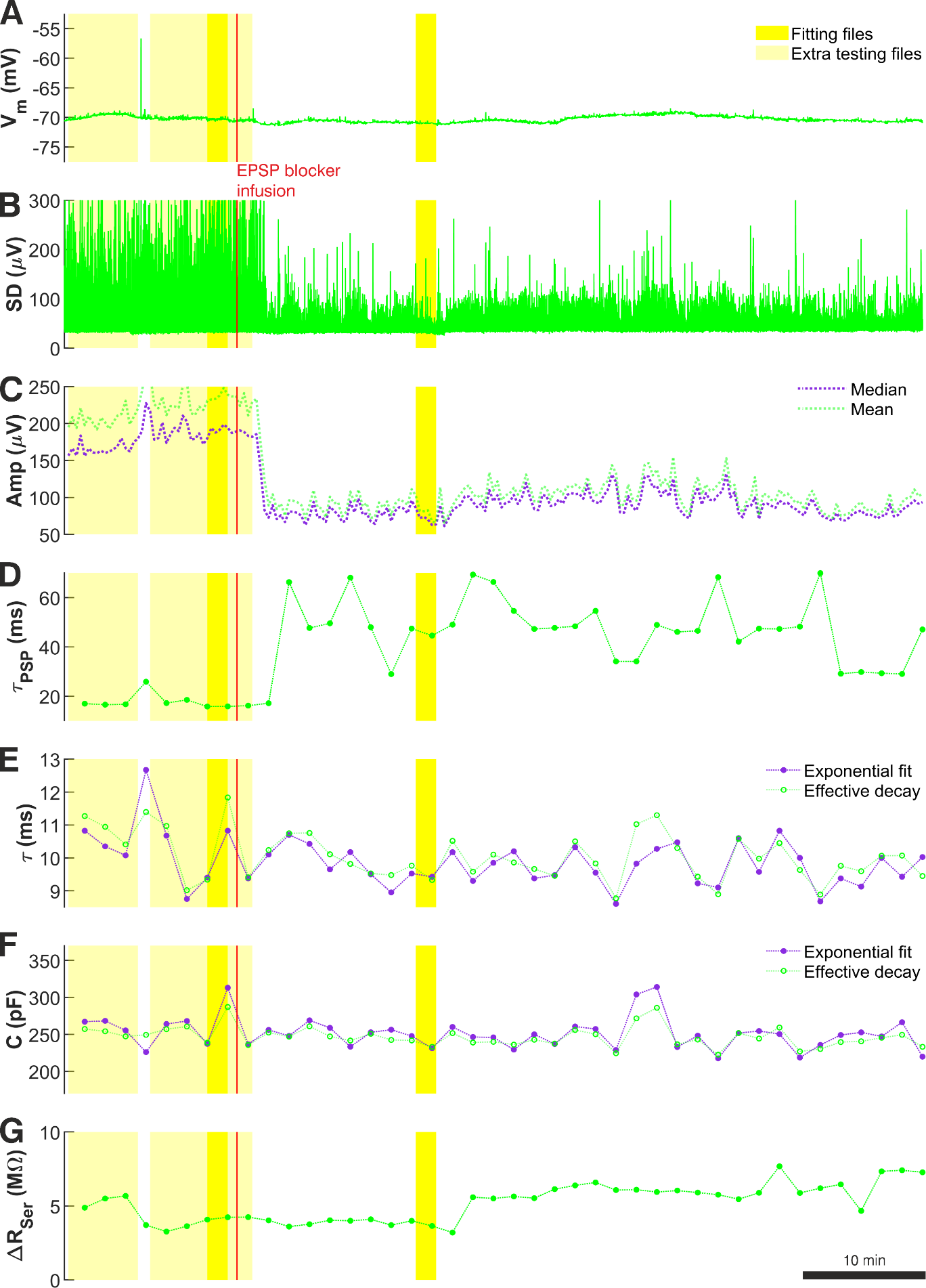
**Supplementary Figure 6:** Cell p122a (layer 2/3). Recording quality measures used to select ’noise with minis’ and ’noise-alone’ sweeps.

Panels A-G as for Figure 2 and Supplementary Figure 2. Data were averaged over 100-second-long windows (5 recording sweeps of 20 s each) in panels D-G; duration 4200 s.


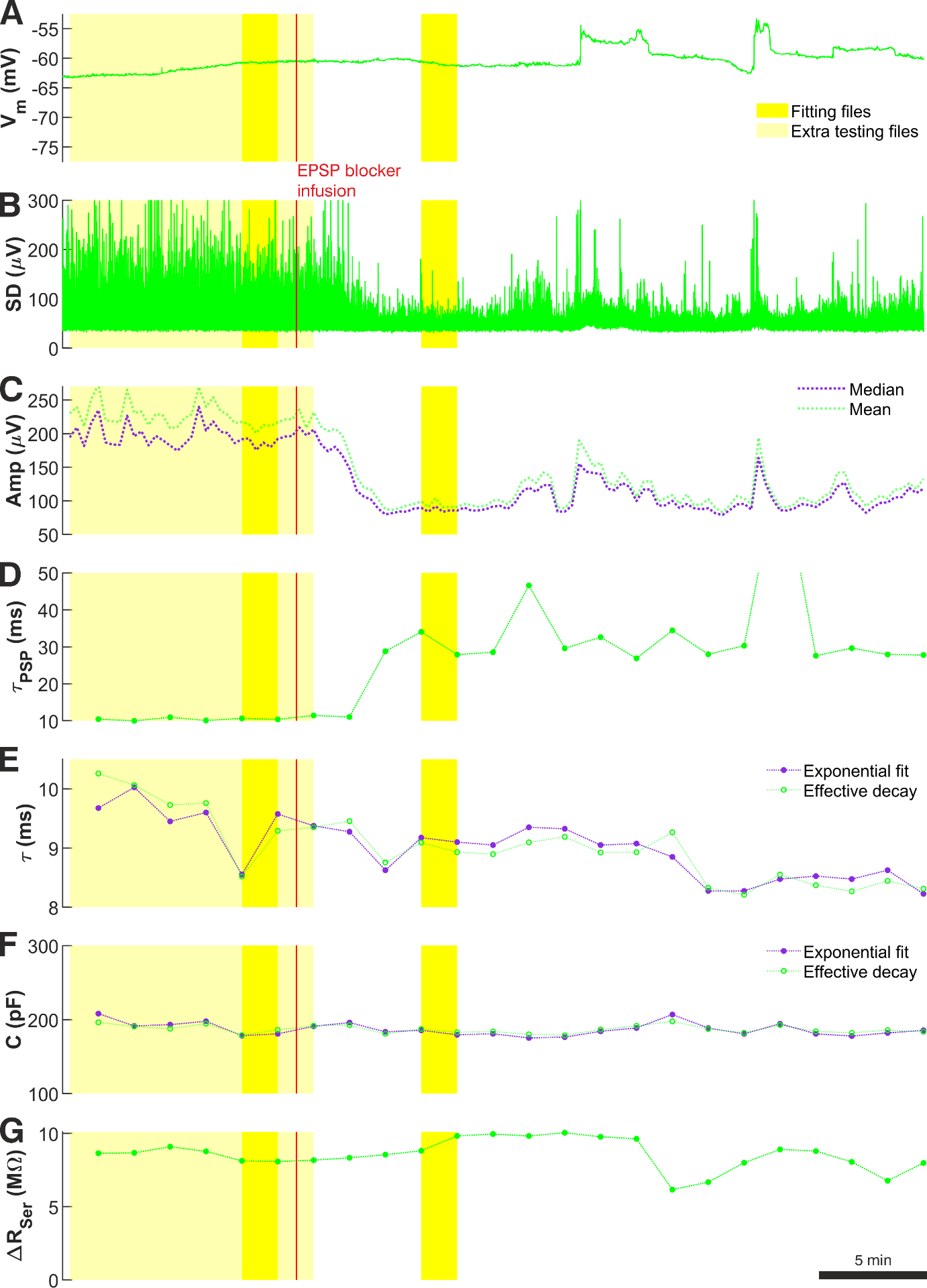
**Supplementary Figure 7:** Cell p124b (layer 5). Recording quality measures used to select ’noise with minis’ and ’noise-alone’ sweeps.

Panels A-G as for Figure 2 and Supplementary Figure 2. Data were averaged over 100-second-long windows (5 recording sweeps of 20 s each) in panels D-G; duration 2400 s.


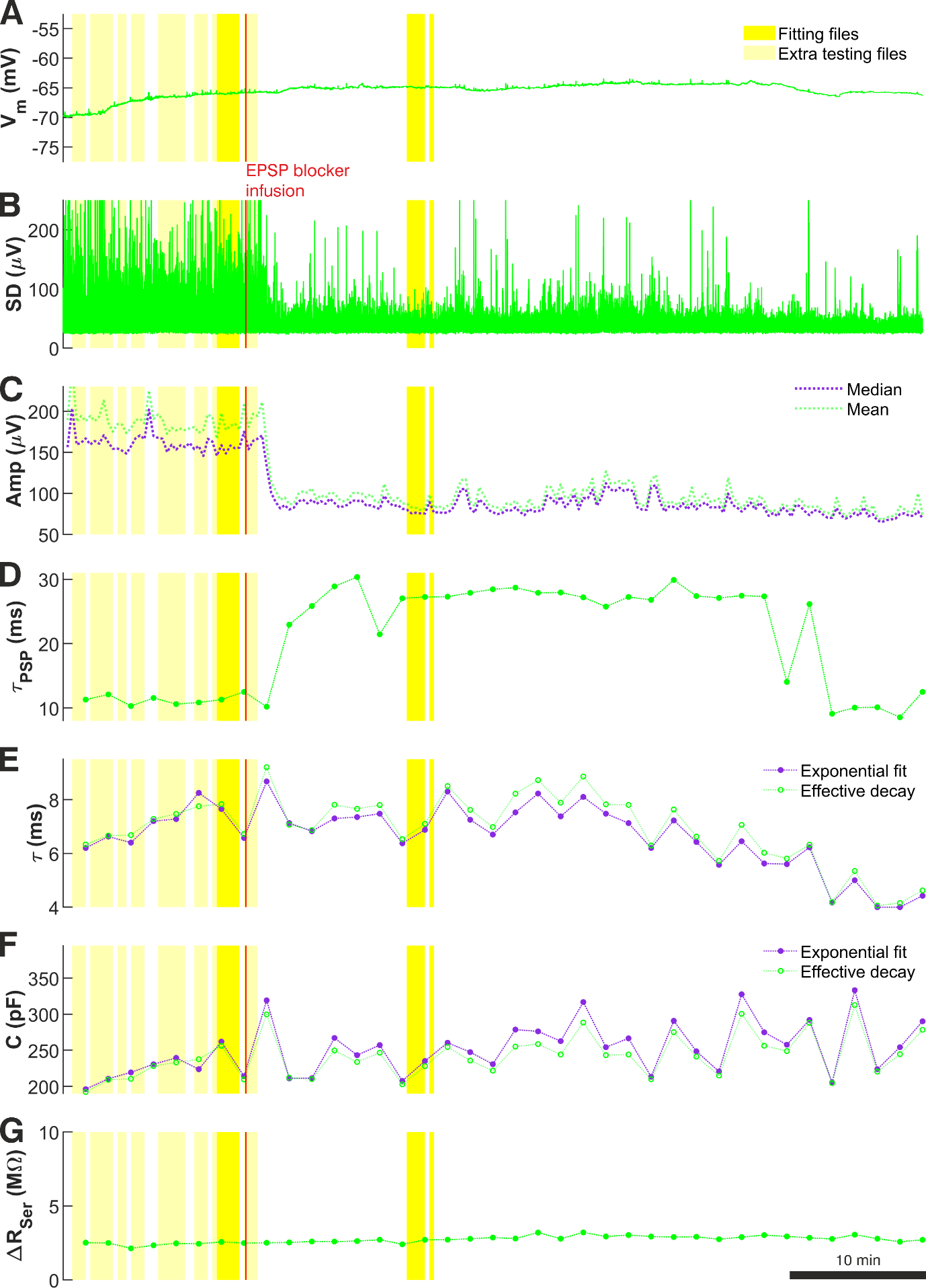
**Supplementary Figure 8:** Cell p125a (layer 2/3). Recording quality measures used to select ’noise with minis’ and ’noise-alone’ sweeps.

Panels A-G as for Figure 2 and Supplementary Figure 2. Data were averaged over 100-second-long windows (5 recording sweeps of 20 s each) in panels D-G; duration 3800 s. Some transiently extra noisy data due to temporary recording instability were excluded from the ‘noise-alone’ epoch.


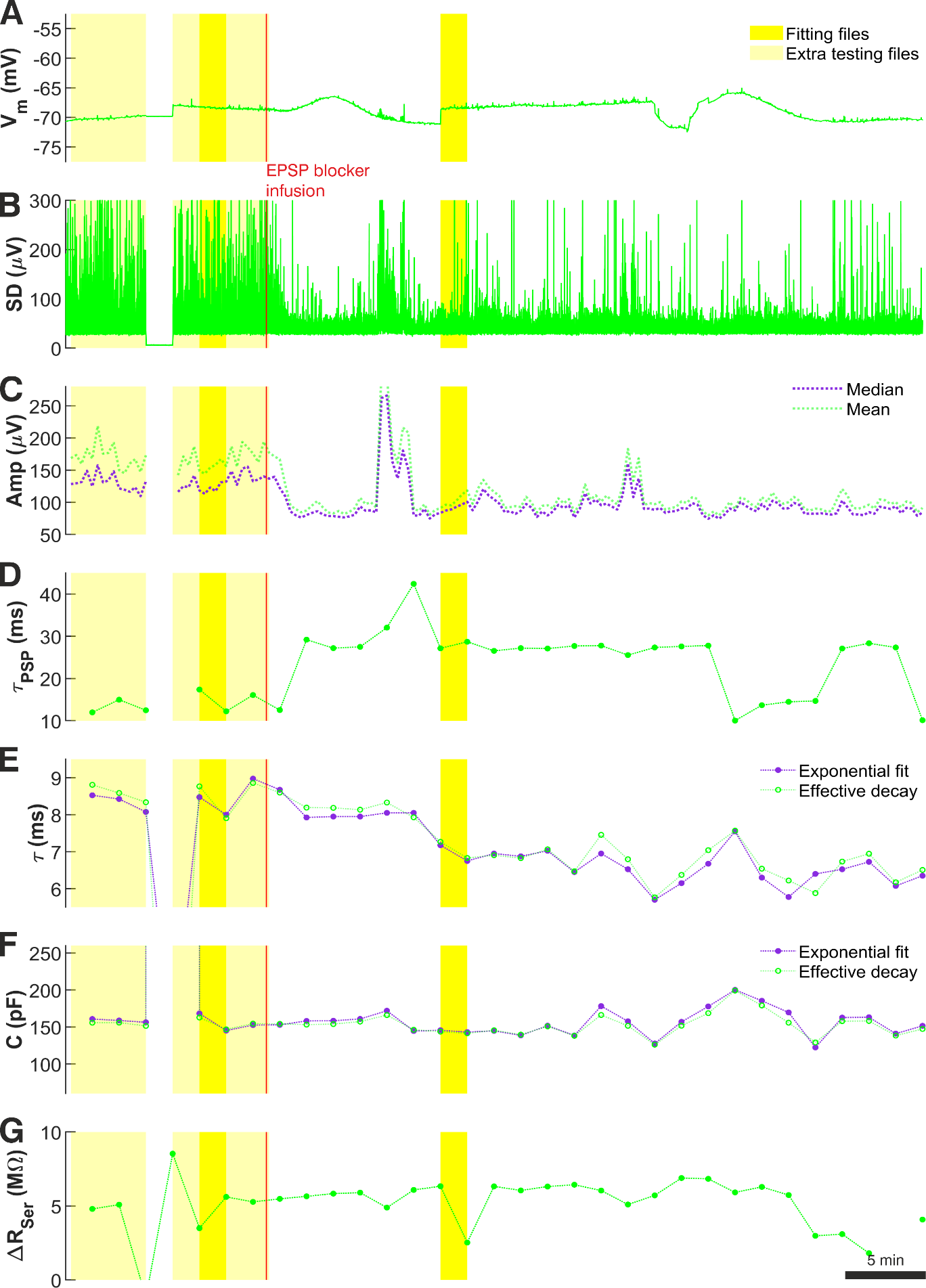
**Supplementary Figure 9:** Cell p127c (layer 2/3). Recording quality measures used to select ’noise with minis’ and ’noise-alone’ sweeps.

Panels A-G as for Figure 2 and Supplementary Figure 2. Data were averaged over 100-second-long windows (5 recording sweeps of 20 s each) in panels D-G; duration 3200 s.


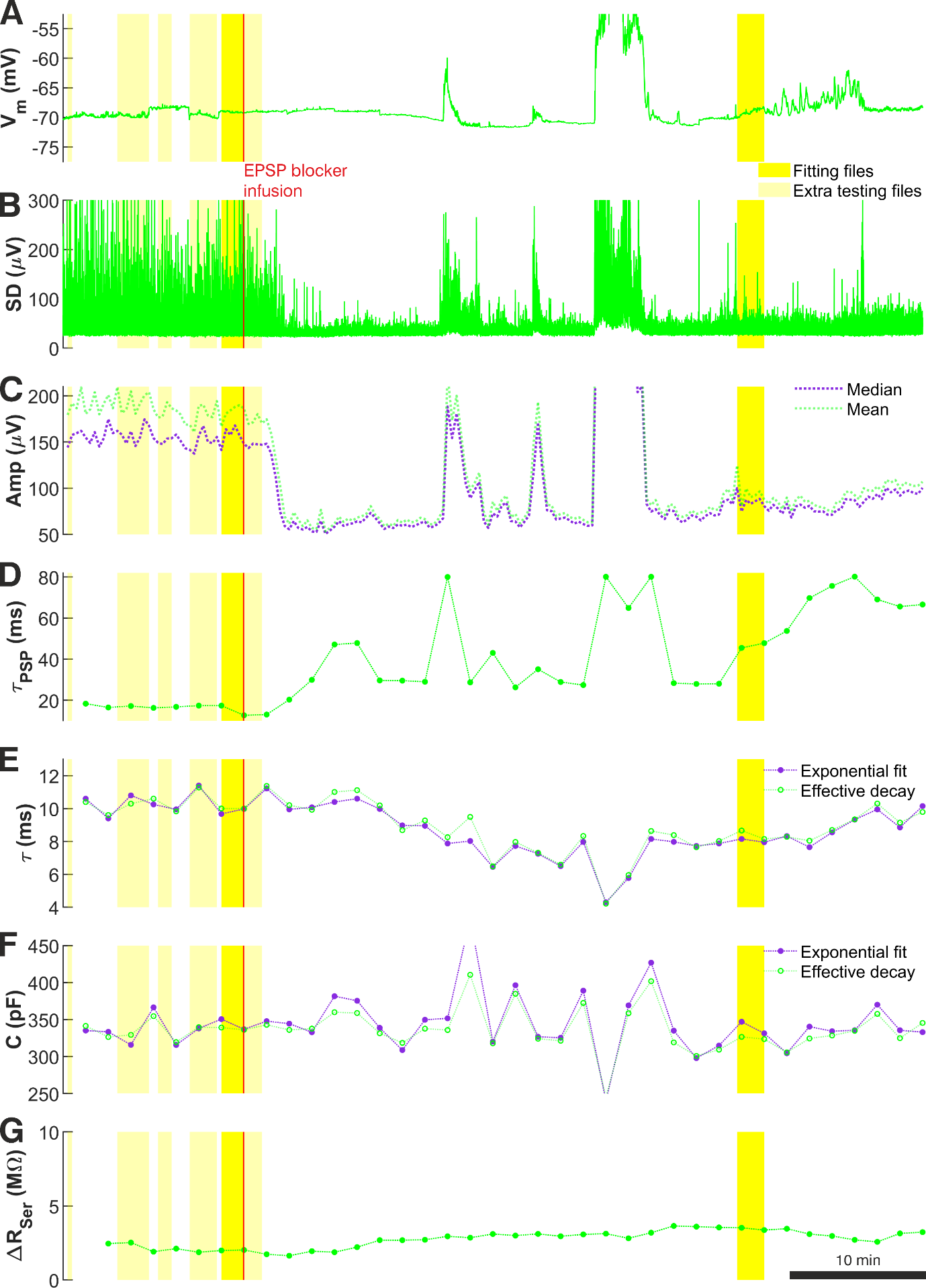


**Supplementary Figure 10:** Cell p128c (layer 2/3). Recording quality measures used to select ’noise with minis’ and ’noise-alone’ sweeps.

Panels A-G as for Figure 2 and Supplementary Figure 2. Data were averaged over 100-second-long windows (5 recording sweeps of 20 s each) in panels D-G; duration 3800 s.


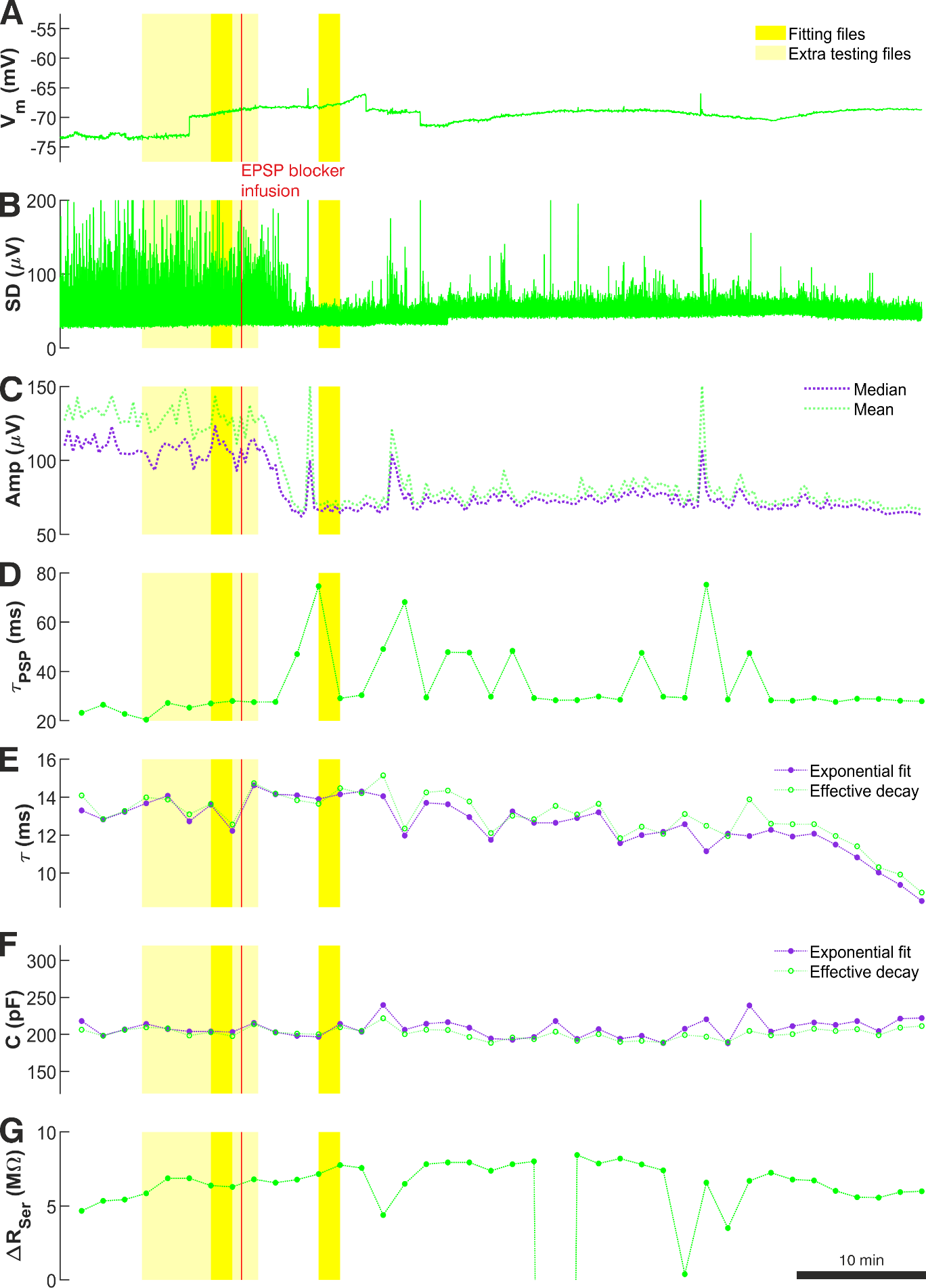
**Supplementary Figure 11:** Cell p129a (layer 5). Recording quality measures used to select ’noise with minis’ and ’noise-alone’ sweeps.

Panels A-G as for Figure 2 and Supplementary Figure 2. Data were averaged over 100-second-long windows (5 recording sweeps of 20 s each) in panels D-G; duration 4000 s.


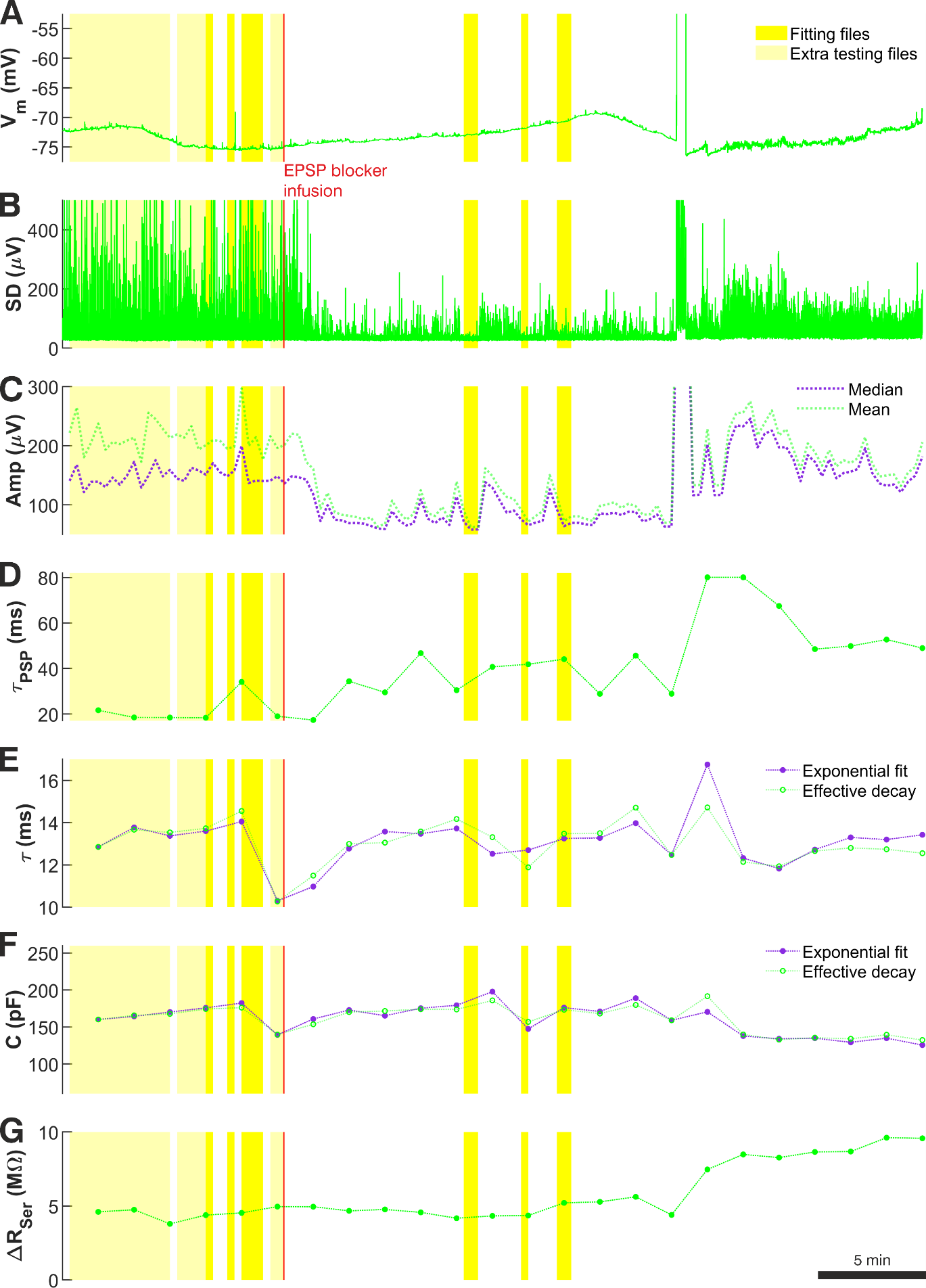
**Supplementary Figure 12:** Cell p131a (layer 2/3). Recording quality measures used to select ’noise with minis’ and ’noise-alone’ sweeps.

Panels A-G as for Figure 2 and Supplementary Figure 2. Data were averaged over 100-second-long windows (5 recording sweeps of 20 s each) in panels D-G; duration 2400 s. Fit epoch is fragmented due to noisy segments, with transient recording instability, being excluded.


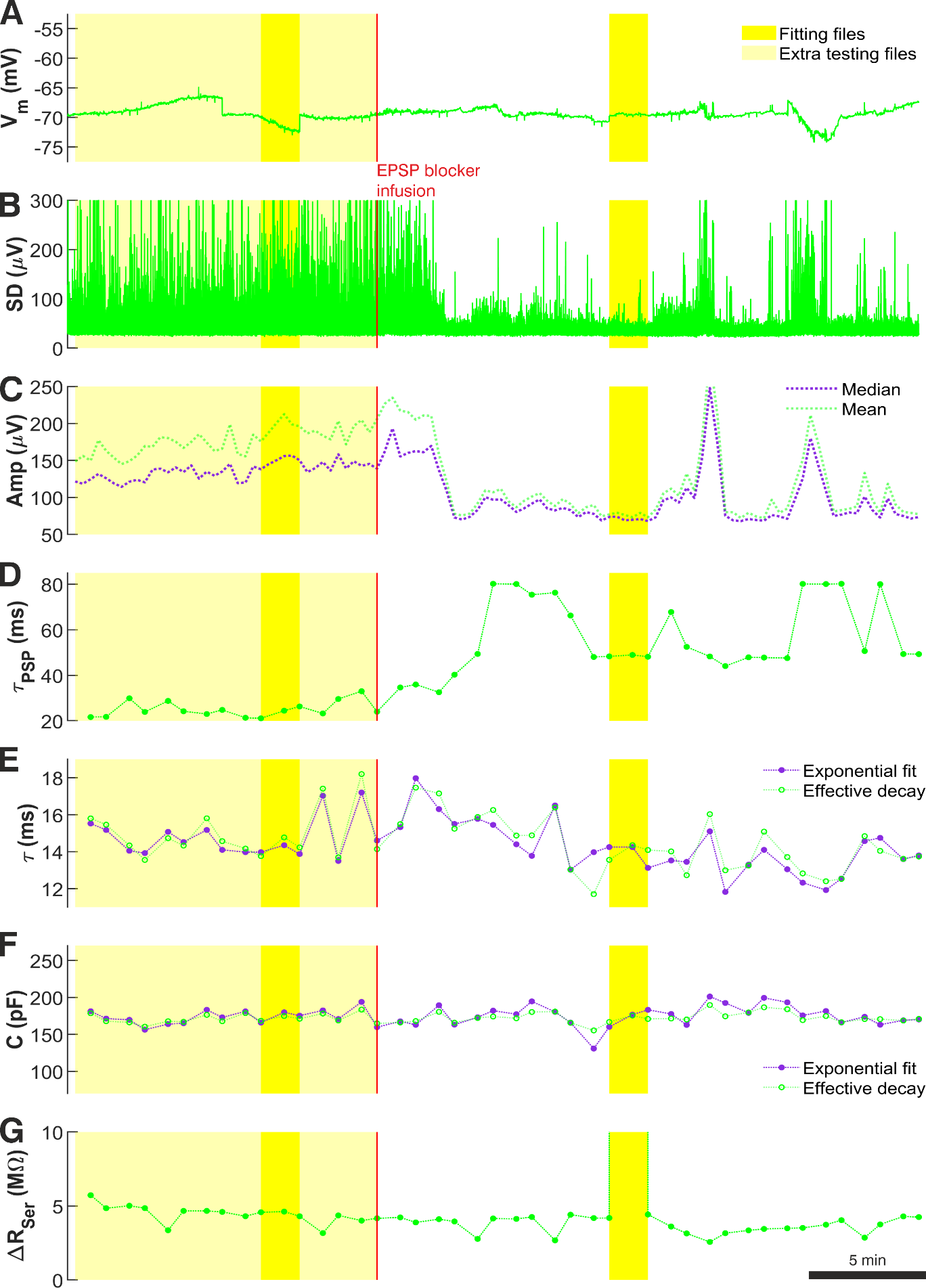
**Supplementary Figure 13:** Cell p131c (layer 2/3). Recording quality measures used to select ’noise with minis’ and ’noise-alone’ sweeps.

Panels A-G as for Figure 2 and Supplementary Figure 2. Data were averaged over 100-second-long windows (5 recording sweeps of 20 s each) in panels D-G; duration 2200 s.


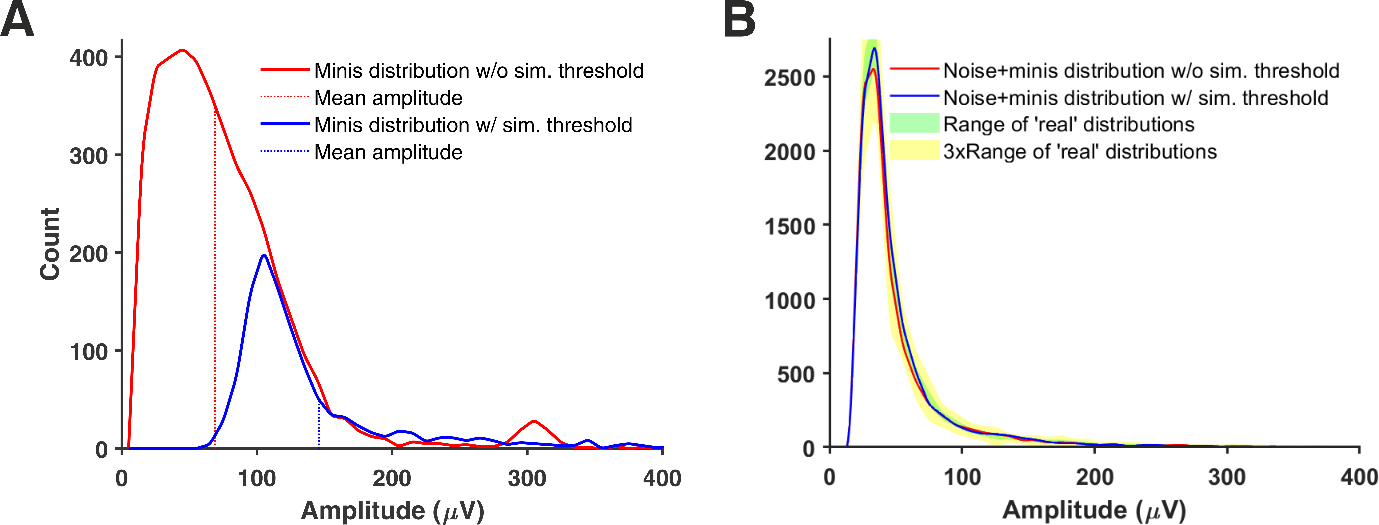


**Supplementary figure 14:** Non-uniqueness of simulated minis distributions fitting the target data.

(A) Two amplitude distributions of simulated minis events generated by the GA using a lower limit on simulated amplitudes of 10 µV (red histogram; termed as without simulation threshold) or 60 µV simulation threshold (blue histogram).

(B) Distributions of mini-like events detected in a ’noise with simulated minis’ voltage traces corresponding to the two minis source distributions in (A). Event histograms are shown superimposed on the full range of ‘real’ (‘noise with real minis’) distributions. The two distributions clearly fall within the range of ‘real’ distributions albeit being generated by two very different source distributions. Recordings obtained from cell p131c (layer 2/3).


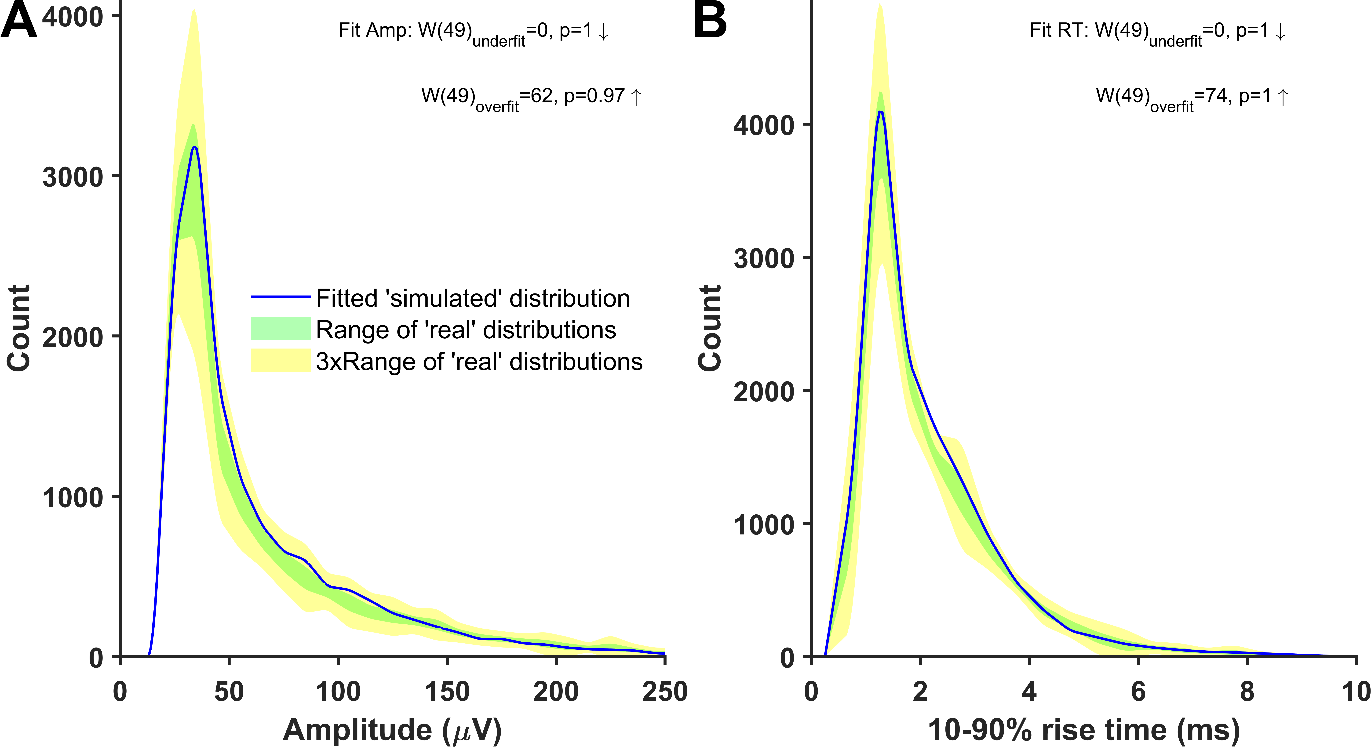
**Supplementary figure 15:** Distributions of mini-like events detected in a ’noise with simulated minis’ voltage trace that closely matched ‘noise with real minis‘ recordings and passed statistical good fit tests, i.e. did not ‘fail’ the poor fit (underfit) unidirectional tests (it could not be ‘picked out’ either objectively or ‘double-blind’ from the ‘noise with real minis’ distributions found with the same detection parameters).

Parameters of simulated minis were controlled by the GA; ’noise-alone’ recording obtained from cell p103a (layer 5). Downward arrows indicate ‘simulated’ SAD < ‘real’ SAD. Upward arrows indicate ‘simulated’ SAD > ‘real’ SAD. The ‘underfit’ (poor fit) unidirectional test (top) involves a comparison with the ‘worst-performing’ (highest between-file) ‘real’ SAD file (most discrepant from the others; the null hypothesis is ‘simulated’ SAD does not exceed ‘real’ SAD). The ‘overfit’ unidirectional test (bottom) involves a comparison with the ‘best-performing’ (lowest between-file) ‘real’ SAD (least discrepant from the others; the null hypothesis is ‘simulated’ SAD is not smaller than ‘real’ SAD), but ‘failure’ of this test (p <0.05 Bonferroni-corrected, difference not by chance) does not indicate a fit rejection.

(A) Amplitude and (B) 10-90% rise time distributions of mini-like events detected in a ’noise with simulated minis’ voltage trace (blue curve).


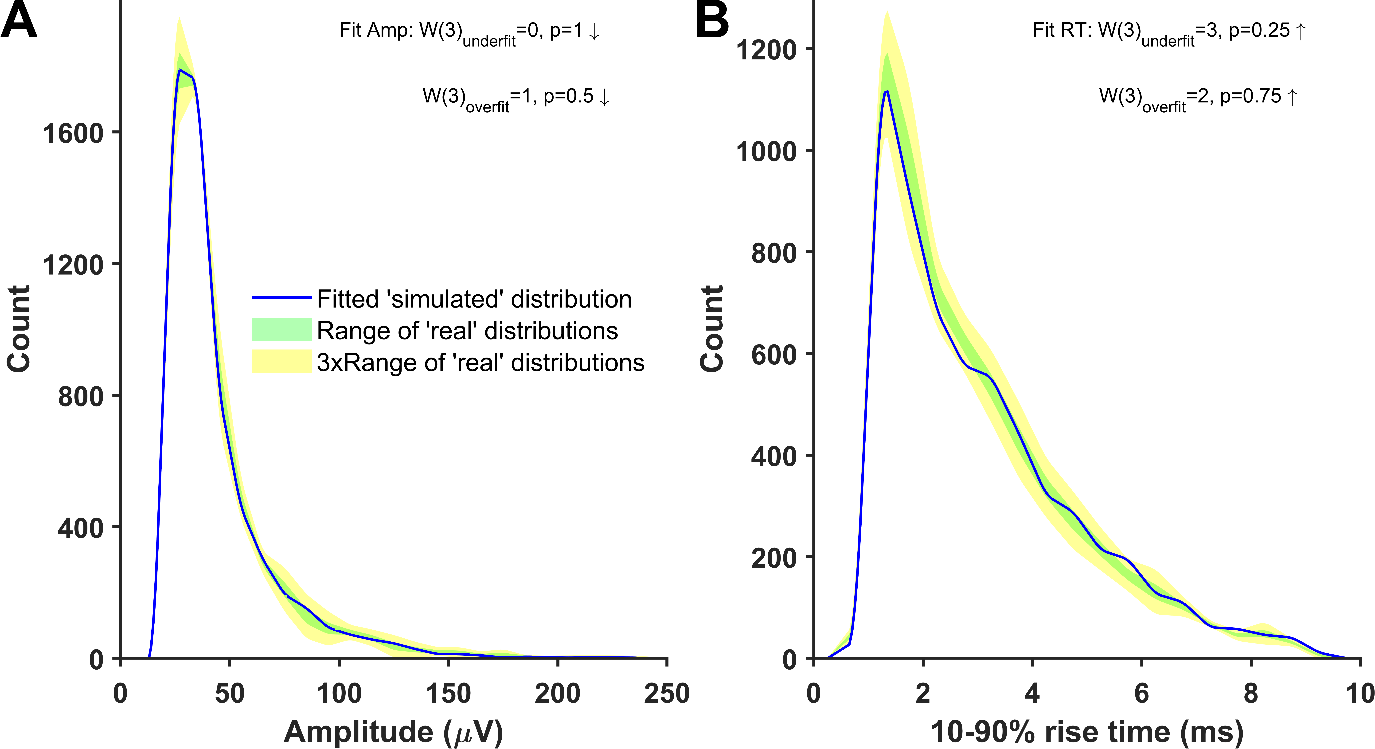
**Supplementary Figure 16:** Cell p108a (layer 5), distributions of mini-like events detected in a ’noise with simulated minis’ voltage trace that closely matched ‘noise with real minis‘ recordings.

Parameters of simulated minis controlled by GA. Down arrow: ‘simulated’ SAD < ‘real’ SAD (up: ‘simulated’ > ‘real’; Suppl. Fig. 15).


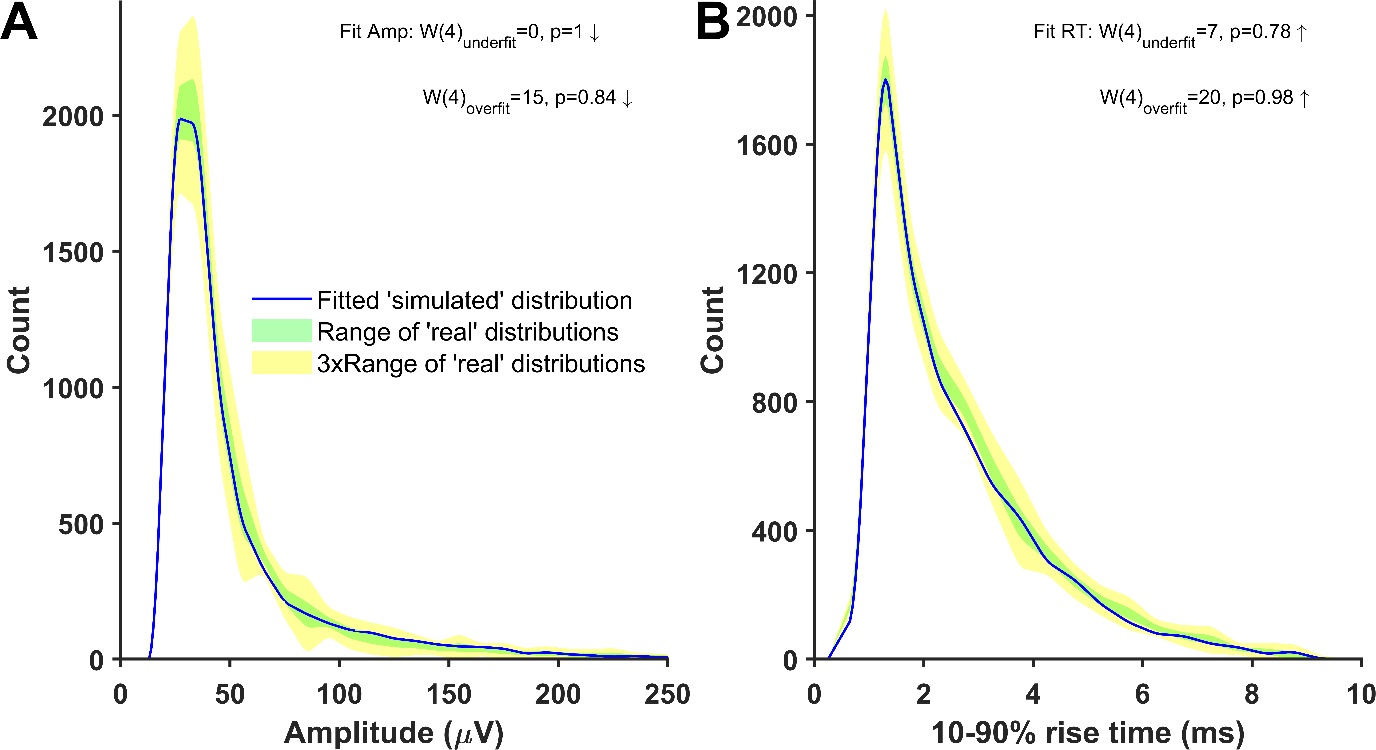
**Supplementary Figure 17:** Cell p108b (layer 5), distributions of mini-like events detected in a ’noise with simulated minis’ voltage trace that closely matched ‘noise with real minis‘ recordings.

Parameters of simulated minis controlled by GA. Down arrow: ‘simulated’ SAD < ‘real’ SAD (up: ‘simulated’ > ‘real’; Suppl. Fig. 15).


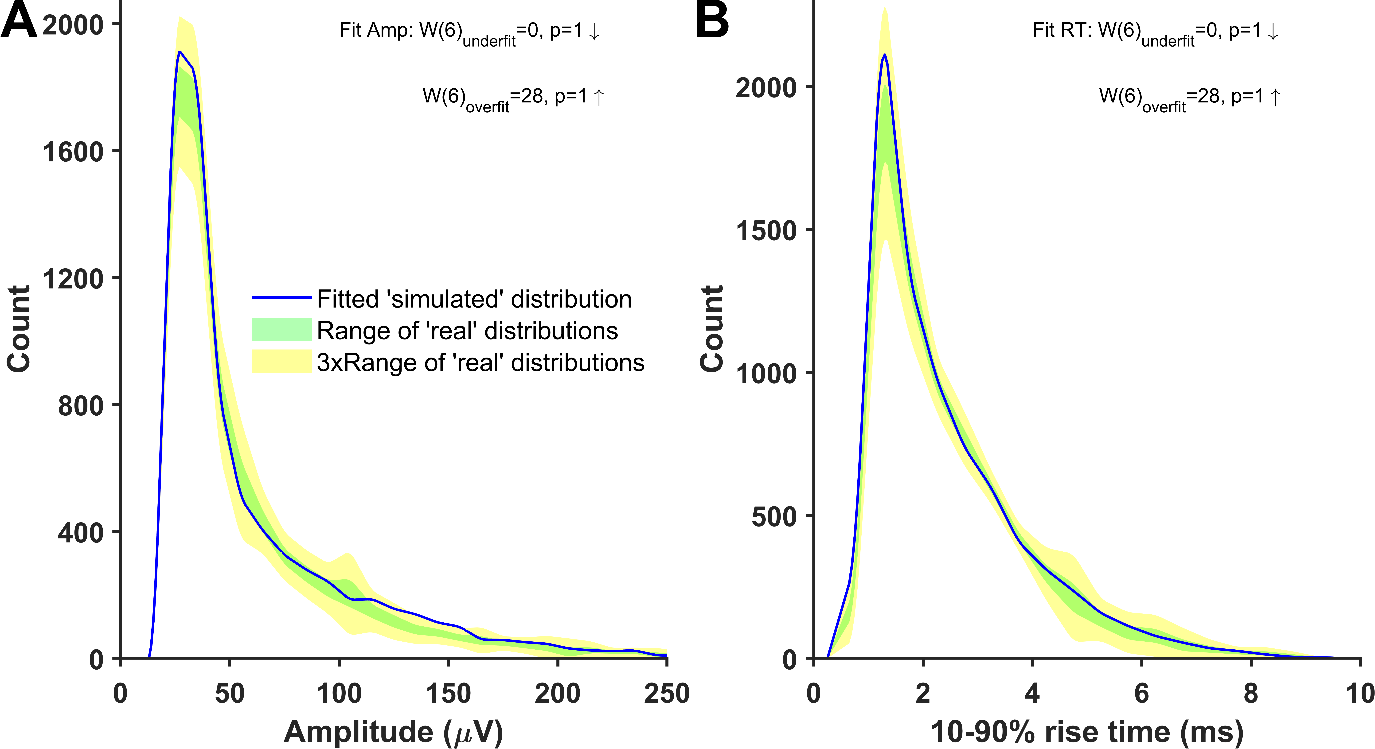
**Supplementary Figure 18:** Cell p108c (layer 5), distributions of mini-like events detected in a ’noise with simulated minis’ voltage trace that closely matched ‘noise with real minis‘ recordings.

Parameters of simulated minis controlled by GA. Down arrow: ‘simulated’ SAD < ‘real’ SAD (up: ‘simulated’ > ‘real’; Suppl. Fig. 15).


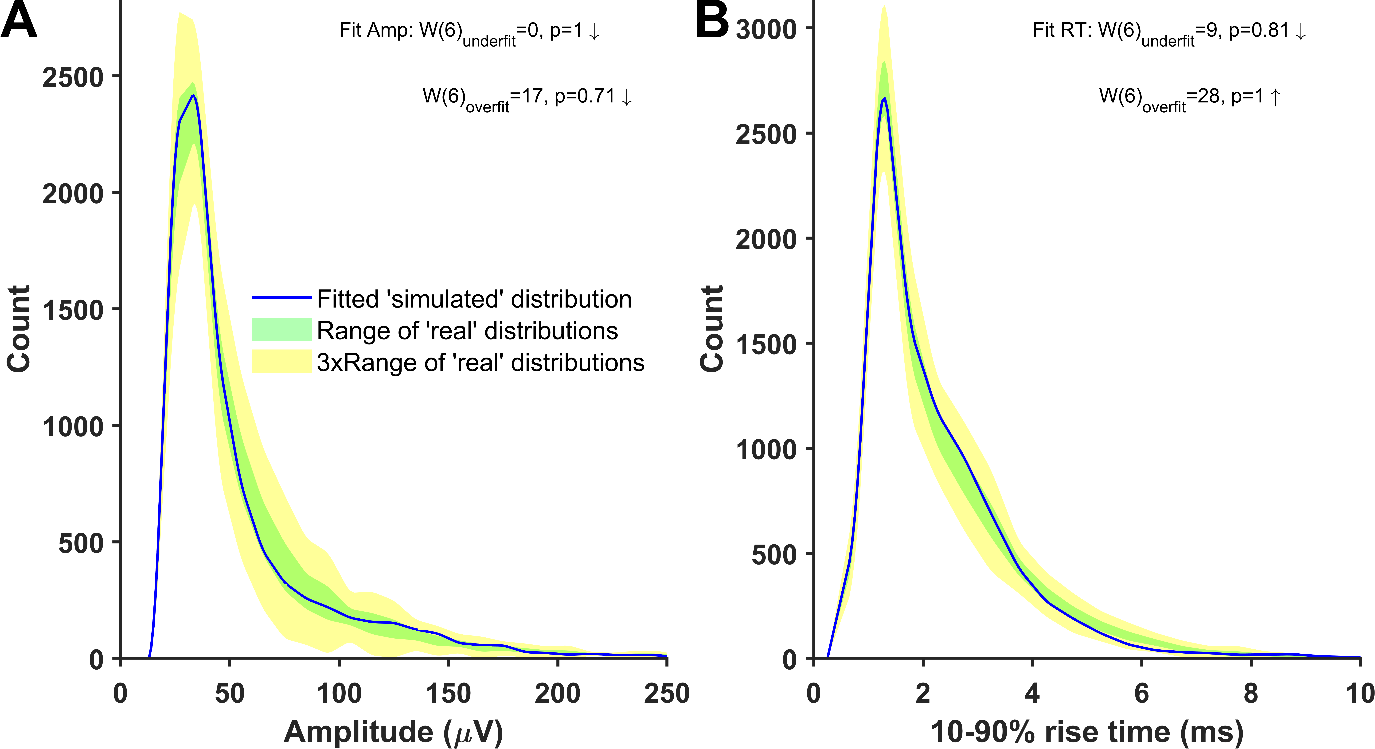
**Supplementary Figure 19:** Cell p120b (layer 2/3), distributions of mini-like events detected in a ’noise with simulated minis’ voltage trace that closely matched ‘noise with real minis‘ recordings.

Parameters of simulated minis controlled by GA. Down arrow: ‘simulated’ SAD < ‘real’ SAD (up: ‘simulated’ > ‘real’; Suppl. Fig. 15).


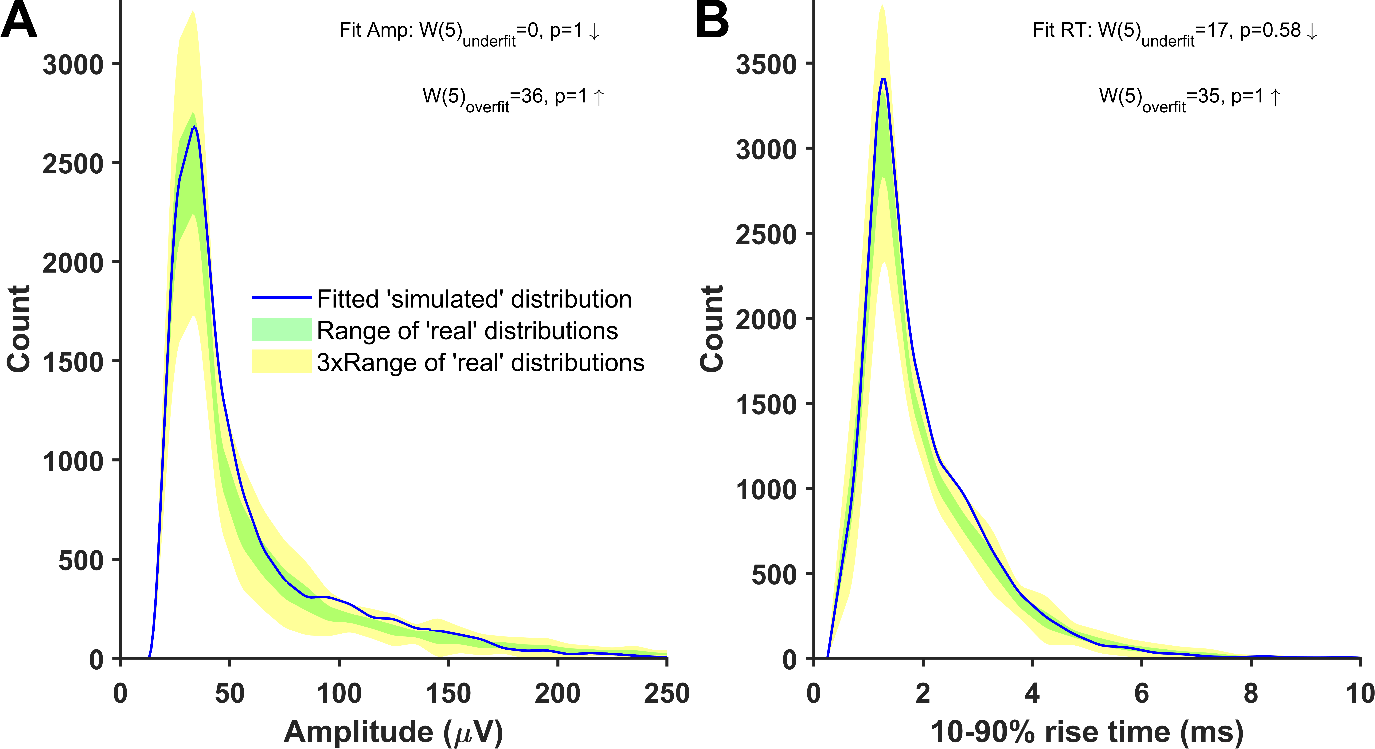
**Supplementary Figure 20:** Cell p122a (layer 2/3), distributions of mini-like events detected in a ’noise with simulated minis’ voltage trace that closely matched ‘noise with real minis‘ recordings.

Parameters of simulated minis controlled by GA. Down arrow: ‘simulated’ SAD < ‘real’ SAD (up: ‘simulated’ > ‘real’; Suppl. Fig. 15).


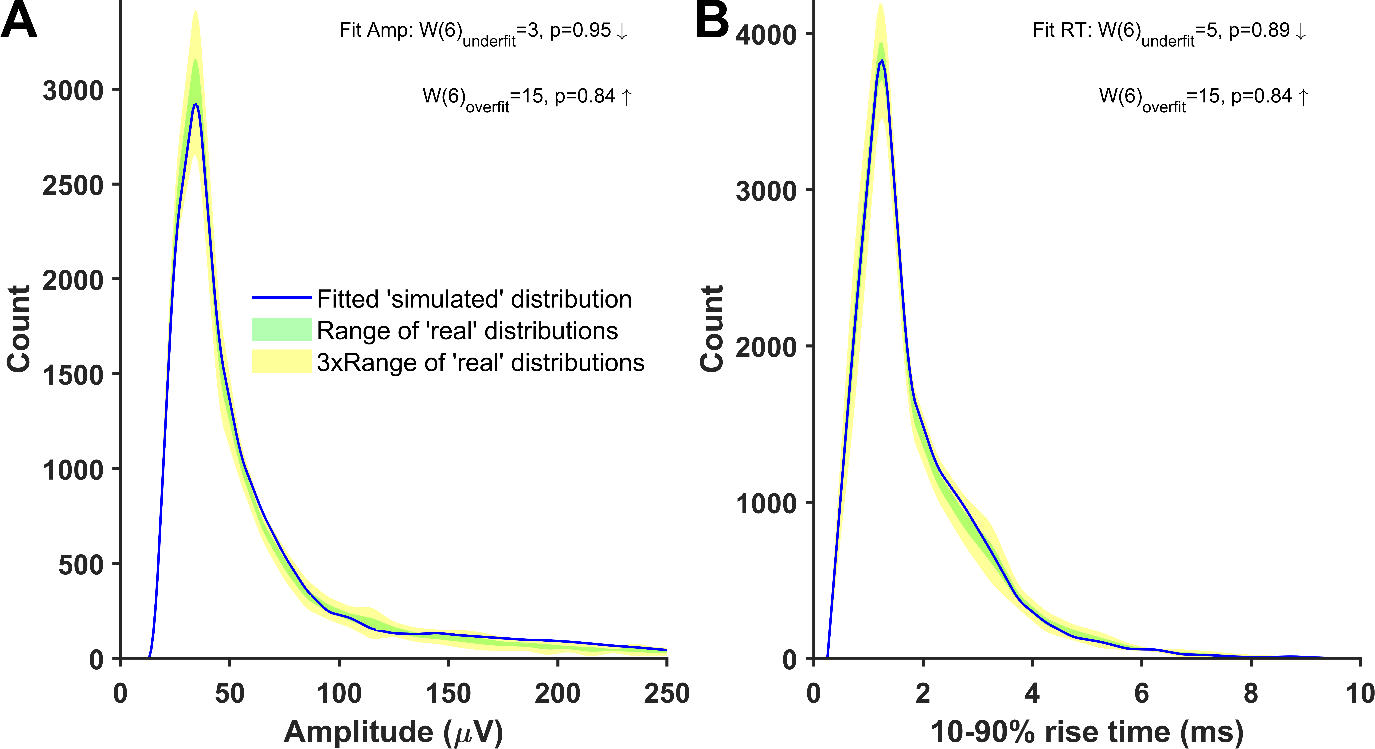
**Supplementary Figure 21:** Cell p124b (layer 5), distributions of mini-like events detected in a ’noise with simulated minis’ voltage trace that closely matched ‘noise with real minis‘ recordings.

Parameters of simulated minis controlled by GA. Down arrow: ‘simulated’ SAD < ‘real’ SAD (up: ‘simulated’ > ‘real’; Suppl. Fig. 15).


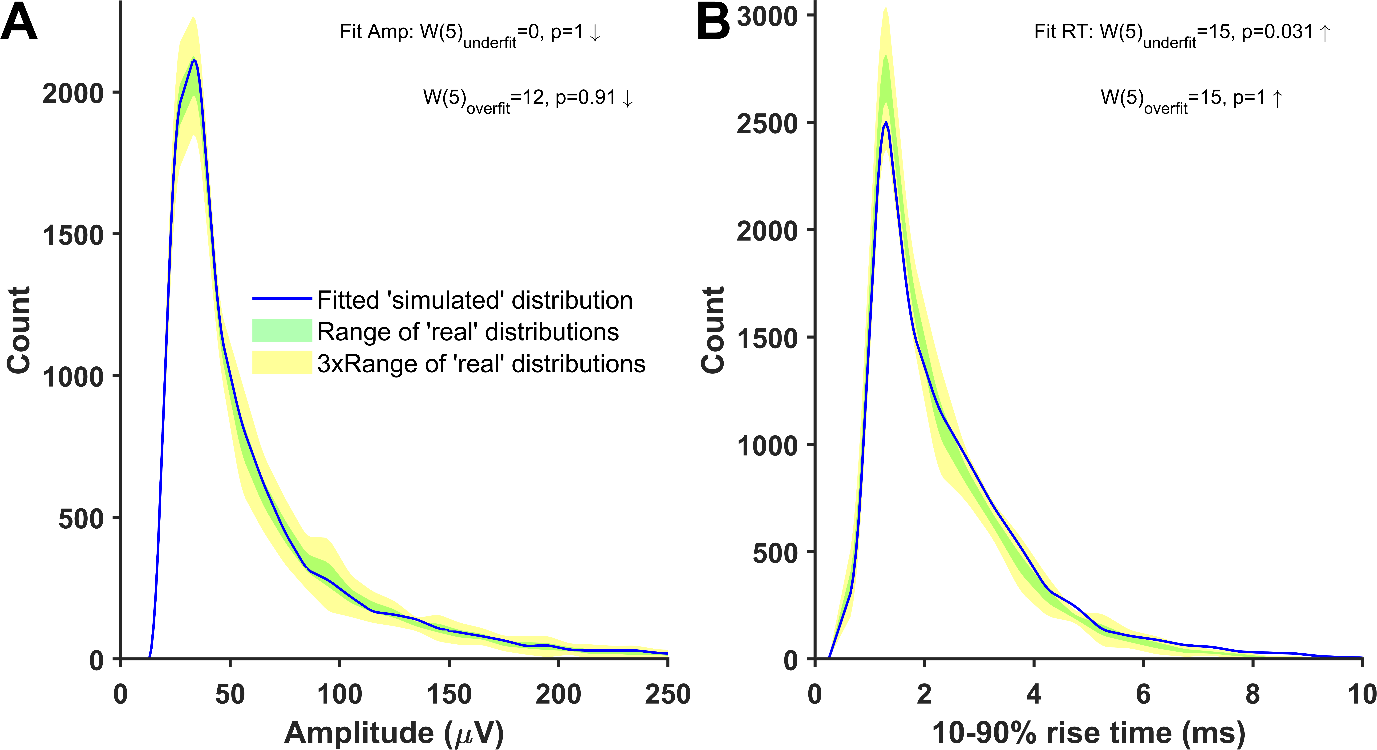
**Supplementary Figure 22:** Cell p125a (layer 2/3), distributions of mini-like events detected in a ’noise with simulated minis’ voltage trace that closely matched ‘noise with real minis‘ recordings.

Parameters of simulated minis controlled by GA. Down arrow: ‘simulated’ SAD < ‘real’ SAD (up: ‘simulated’ > ‘real’; Suppl. Fig. 15).


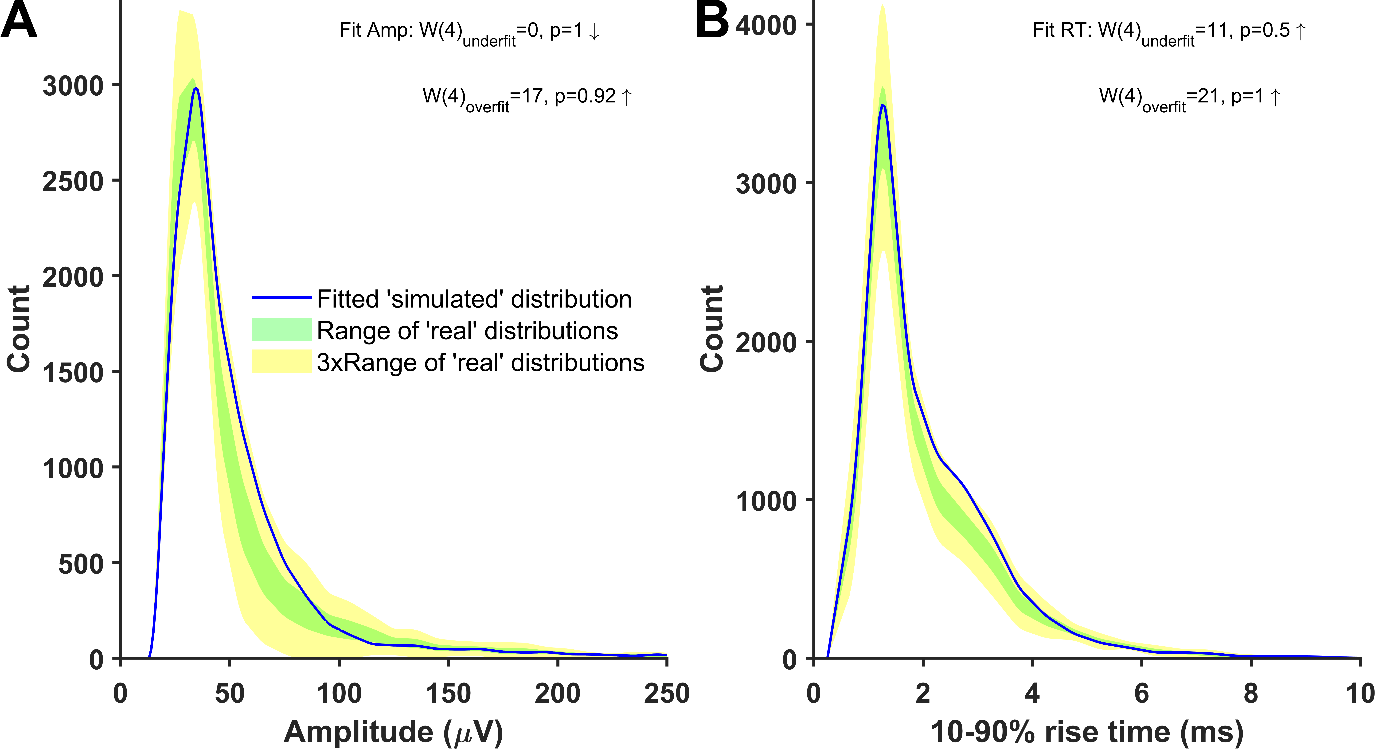
**Supplementary Figure 23:** Cell p127c (layer 2/3), distributions of mini-like events detected in a ’noise with simulated minis’ voltage trace that closely matched ‘noise with real minis‘ recordings.

Parameters of simulated minis controlled by GA. Down arrow: ‘simulated’ SAD < ‘real’ SAD (up: ‘simulated’ > ‘real’; Suppl. Fig. 15).


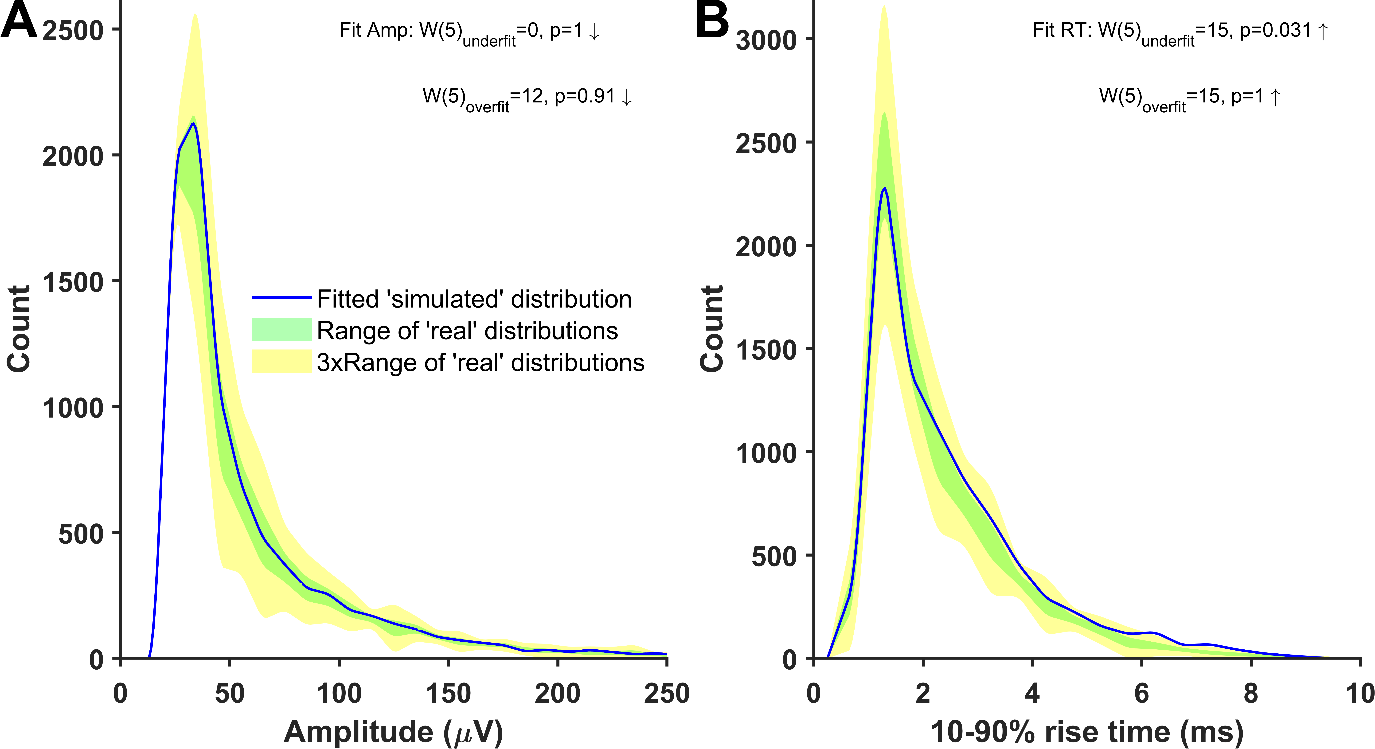
**Supplementary Figure 24:** Cell p128c (layer 2/3), distributions of mini-like events detected in a ’noise with simulated minis’ voltage trace that closely matched ‘noise with real minis‘ recordings.

Parameters of simulated minis controlled by GA. Down arrow: ‘simulated’ SAD < ‘real’ SAD (up: ‘simulated’ > ‘real’; Suppl. Fig. 15).


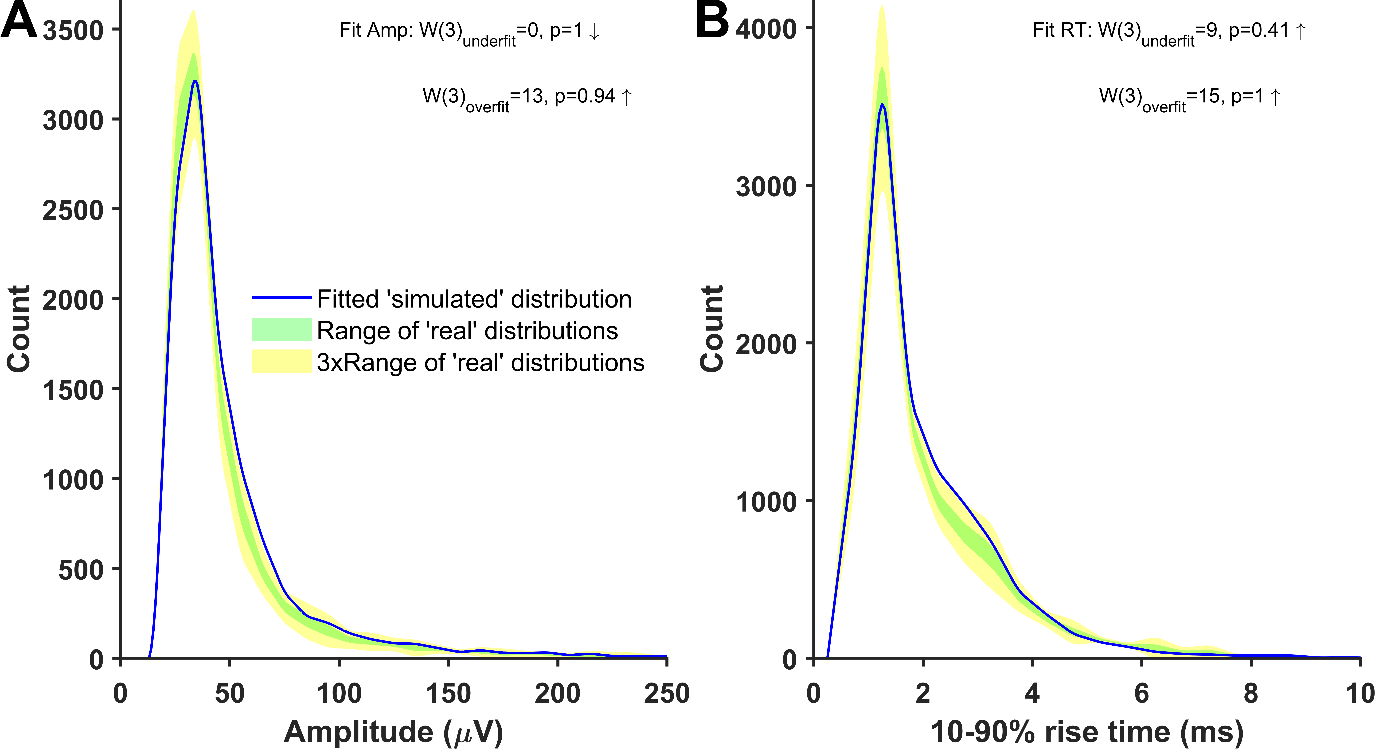
**Supplementary Figure 25:** Cell p129a (layer 5), distributions of mini-like events detected in a ’noise with simulated minis’ voltage trace that closely matched ‘noise with real minis‘ recordings.

Parameters of simulated minis controlled by GA. Down arrow: ‘simulated’ SAD < ‘real’ SAD (up: ‘simulated’ > ‘real’; Suppl. Fig. 15).


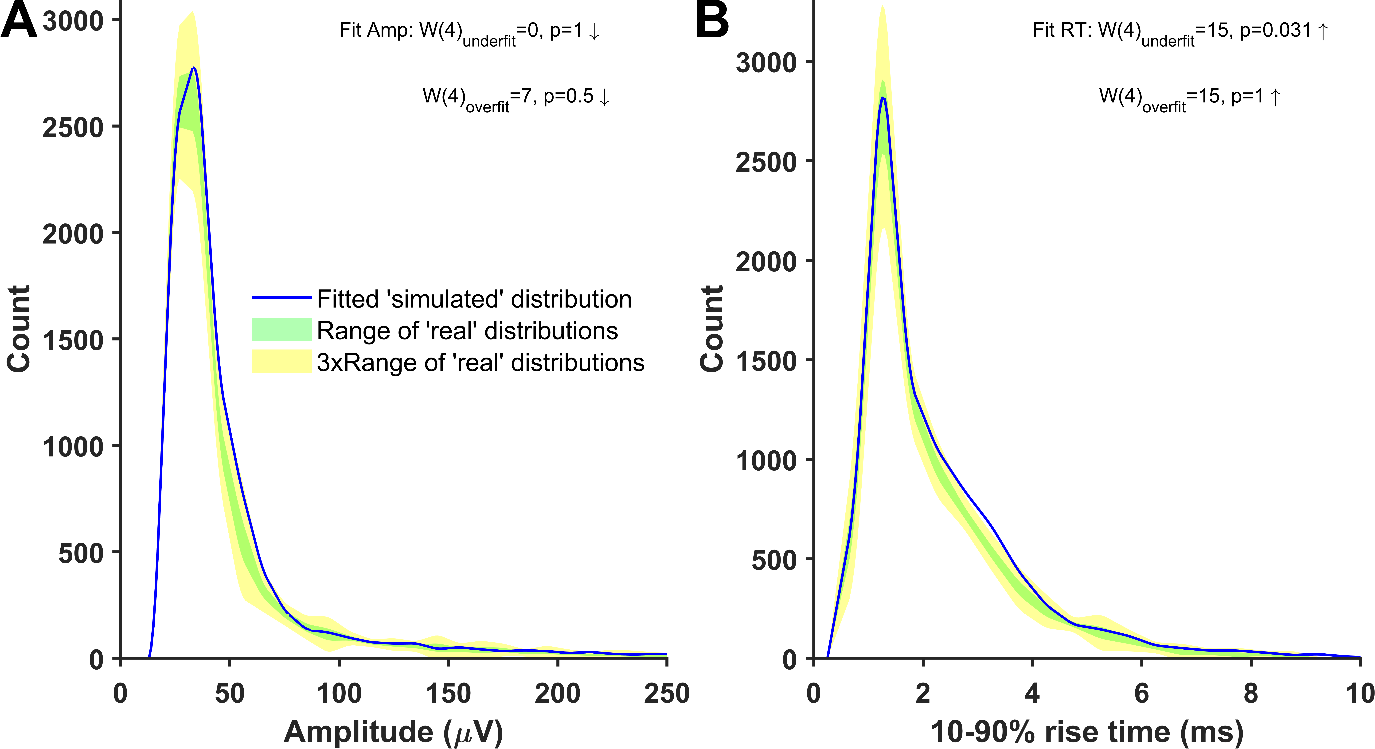
**Supplementary Figure 26:** Cell p131a (layer 2/3), distributions of mini-like events detected in a ’noise with simulated minis’ voltage trace that closely matched ‘noise with real minis‘ recordings.

Parameters of simulated minis controlled by GA. Down arrow: ‘simulated’ SAD < ‘real’ SAD (up: ‘simulated’ > ‘real’; Suppl. Fig. 15).


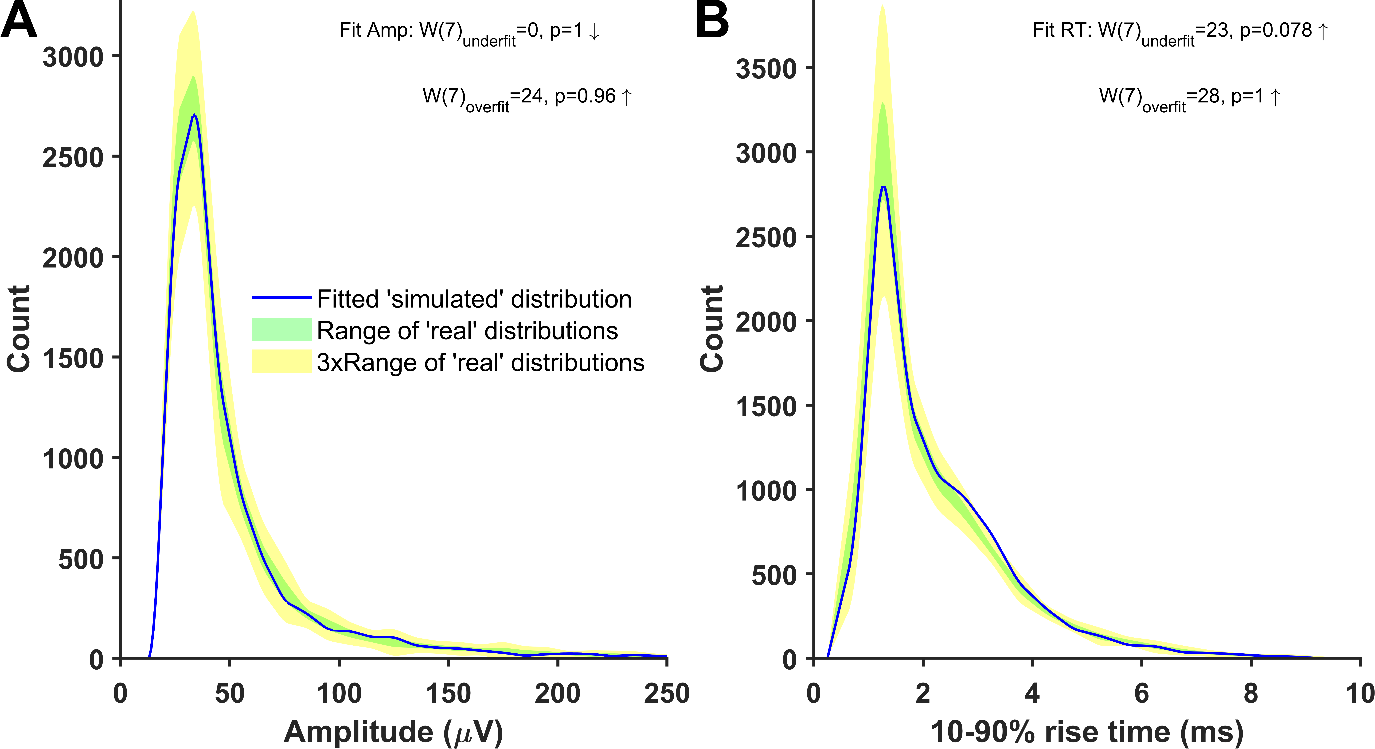
**Supplementary Figure 27:** Cell p131c (layer 2/3), distributions of mini-like events detected in a ’noise with simulated minis’ voltage trace that closely matched ‘noise with real minis‘ recordings.

Parameters of simulated minis controlled by GA. Down arrow: ‘simulated’ SAD < ‘real’ SAD (up: ‘simulated’ > ‘real’; Suppl. Fig. 15).


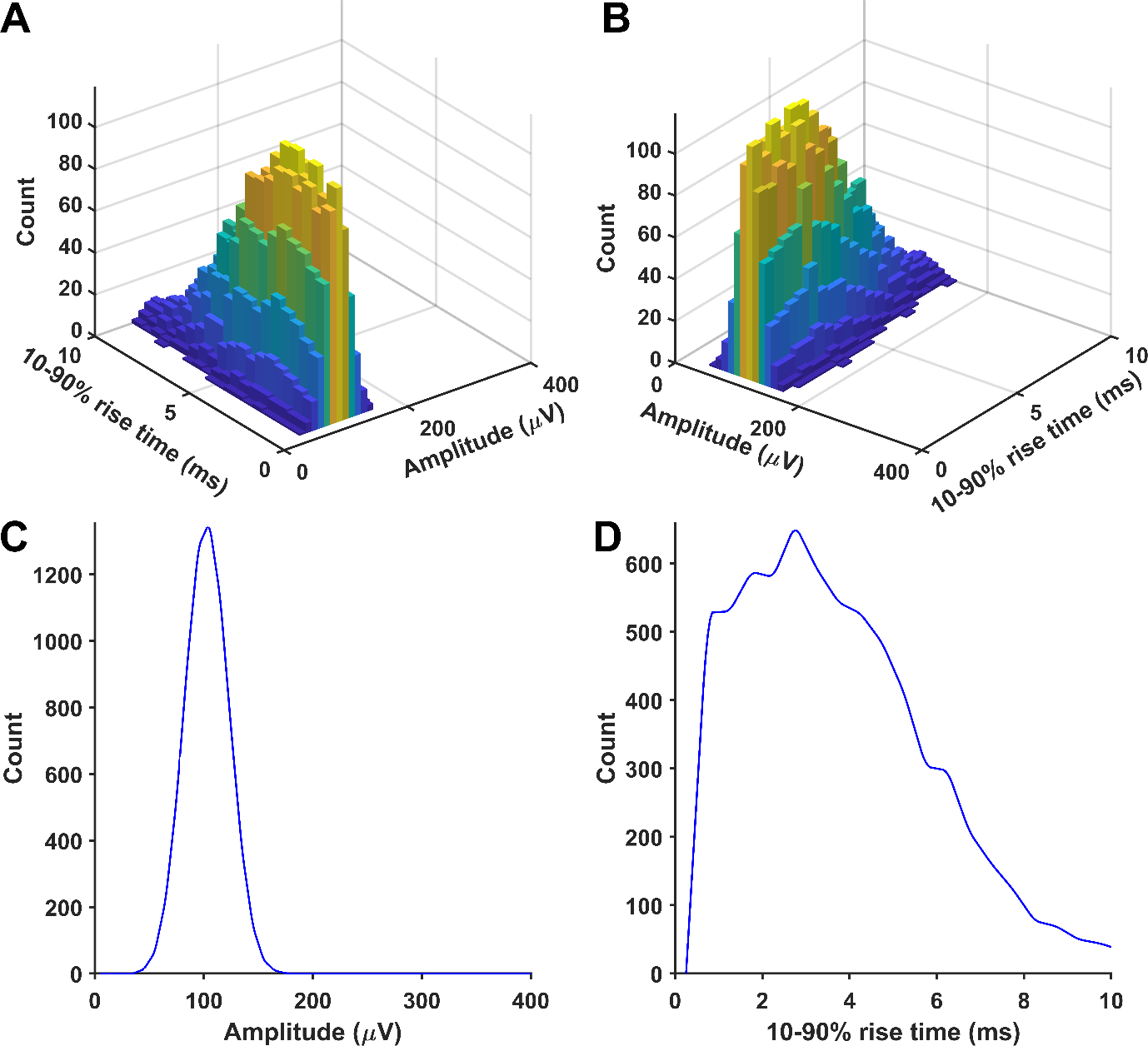
**Supplementary Figure 28:** An example of a distribution of randomly selected simulated minis that was used to produce a close match between distributions of mini-like events detected in a ’noise with simulated minis’ voltage trace (40 µV lower limit on simulated amplitudes) and a ’noise with real minis’ recording for cell p103a (layer 5).

(A) and (B) Joint amplitude and 10-90% rise time distribution of simulated minis (two views).

(C) ‘Marginal’ amplitude and (D) 10-90% rise time distributions (all 2-D bins projected onto that axis).


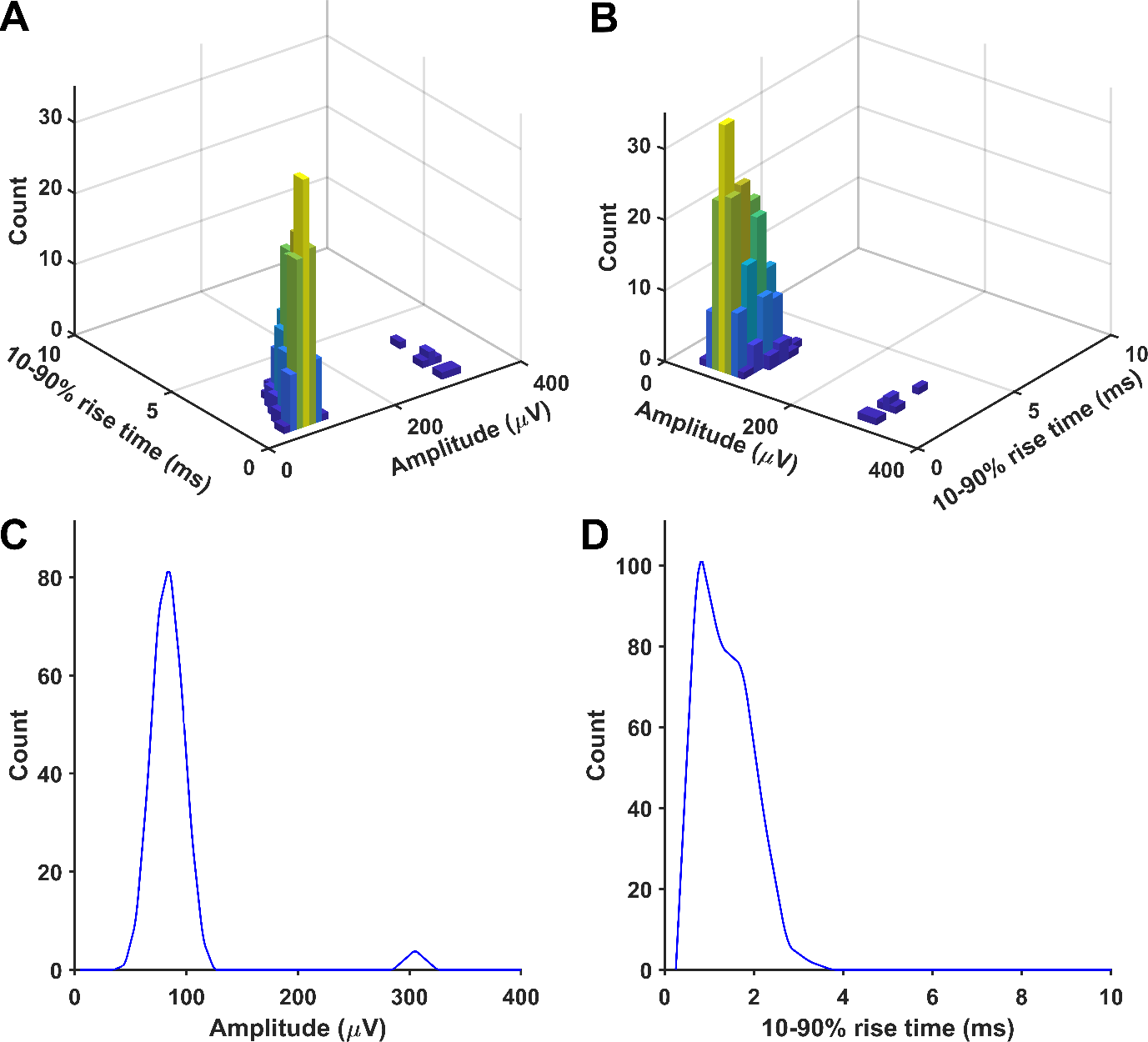
**Supplementary Figure 29:** An example of a distribution of randomly selected simulated minis that was used to produce a close match between distributions of mini-like events detected in a ’noise with simulated minis’ voltage trace (40 µV lower limit on simulated amplitudes) and a ’noise with real minis’ recording for cell p106b (layer 5).

Panels as for Supplementary Figure 28.


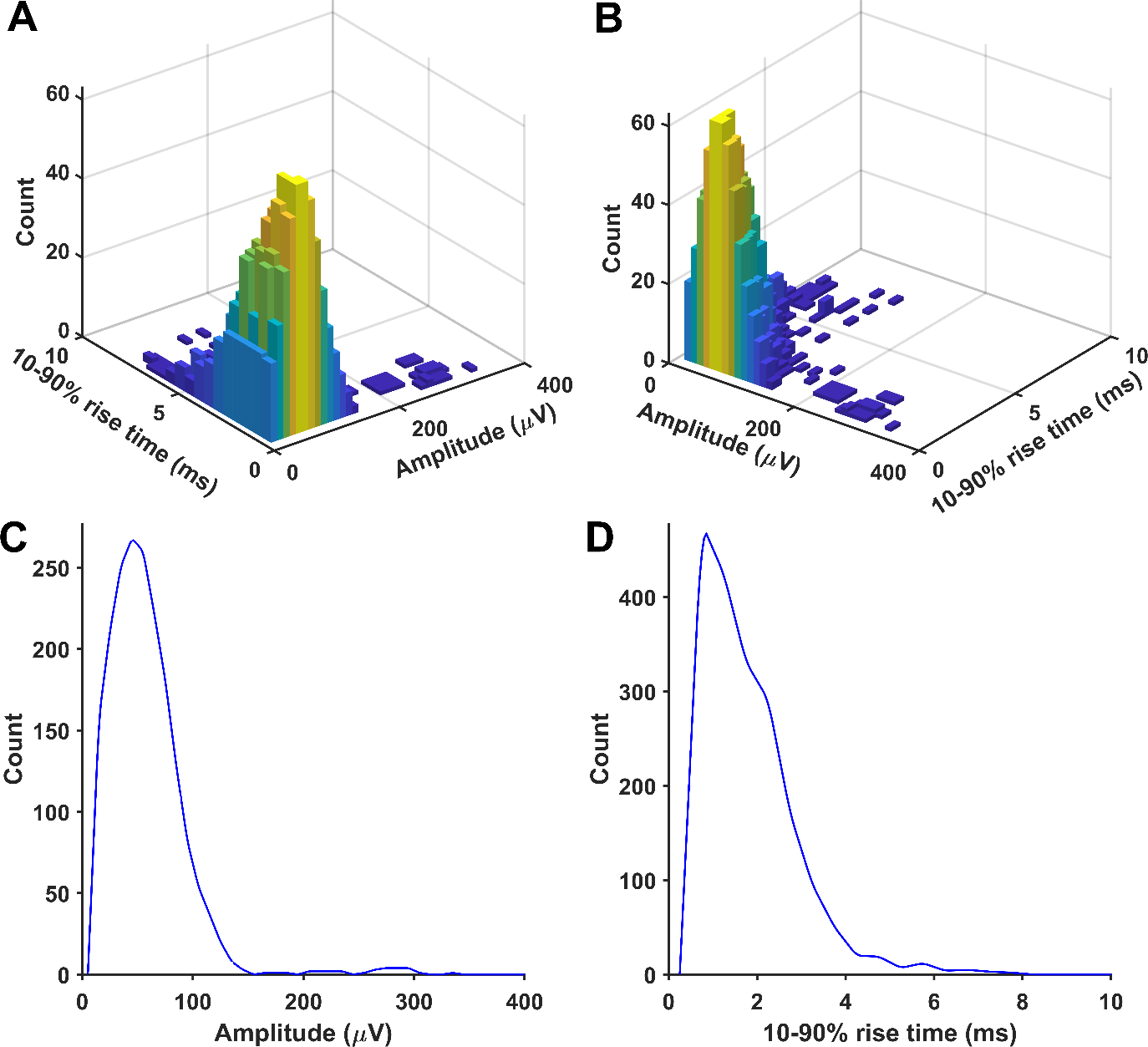
**Supplementary Figure 30:** An example of a distribution of randomly selected simulated minis that was used to produce a close match between distributions of mini-like events detected in a ’noise with simulated minis’ voltage trace (10 µV lower limit on simulated amplitudes) and a ’noise with real minis’ recording for cell p108a (layer 5).

Panels as for Supplementary Figure 28.


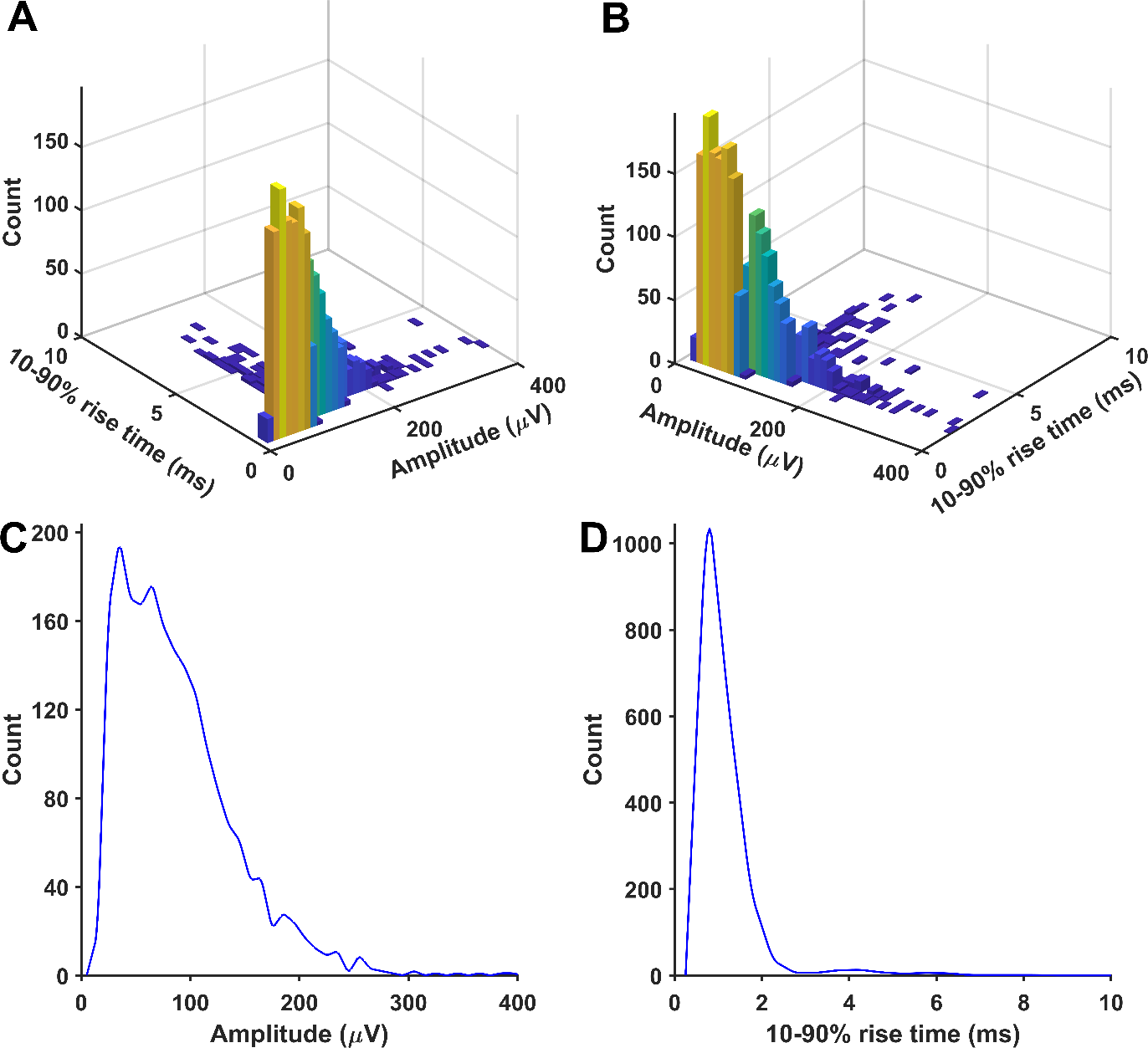
**Supplementary Figure 31:** An example of a distribution of randomly selected simulated minis that was used to produce a close match between distributions of mini-like events detected in a ’noise with simulated minis’ voltage trace (10 µV lower limit on simulated amplitudes) and a ’noise with real minis’ recording for cell p108b (layer 5).

Panels as for Supplementary Figure 28.


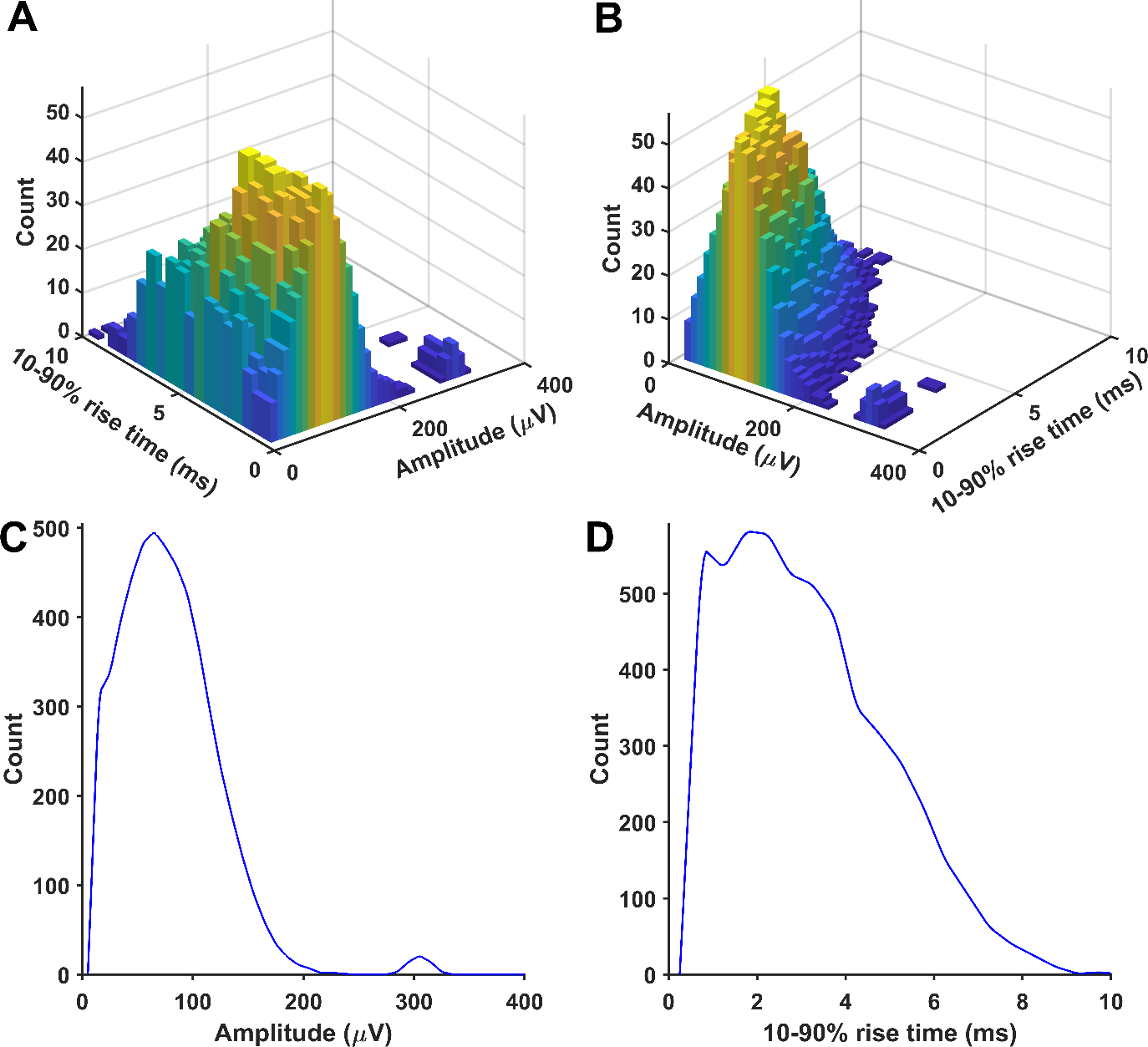
**Supplementary Figure 32:** An example of a distribution of randomly selected simulated minis that was used to produce a close match between distributions of mini-like events detected in a ’noise with simulated minis’ voltage trace (10 µV lower limit on simulated amplitudes) and a ’noise with real minis’ recording for cell p108c (layer 5).

Panels as for Supplementary Figure 28.


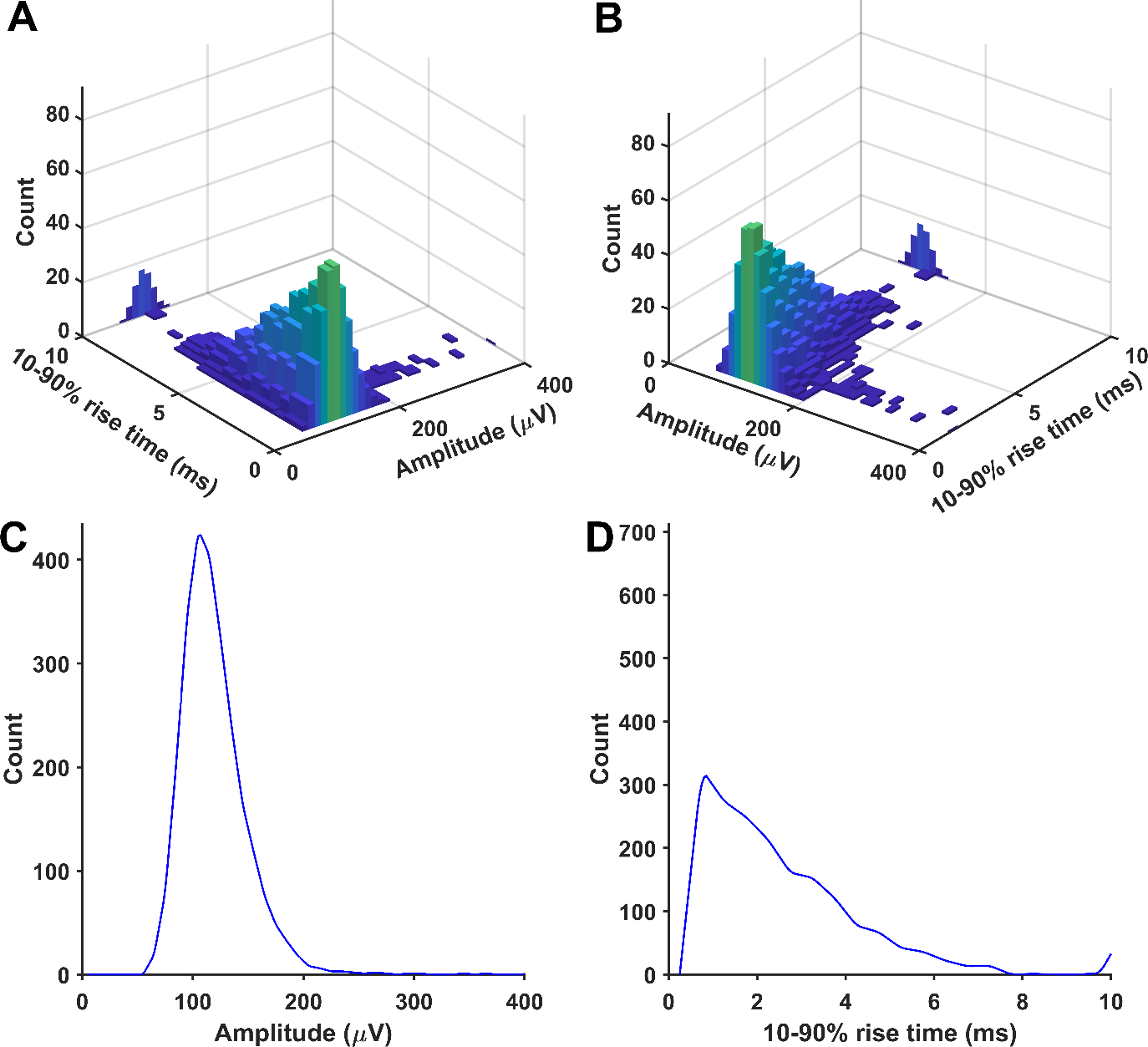
**Supplementary Figure 33:** An example of a distribution of randomly selected simulated minis that was used to produce a close match between distributions of mini-like events detected in a ’noise with simulated minis’ voltage trace (60 µV lower limit on simulated amplitudes) and a ’noise with real minis’ recording for cell p120b (layer 2/3).

Panels as for Supplementary Figure 28.


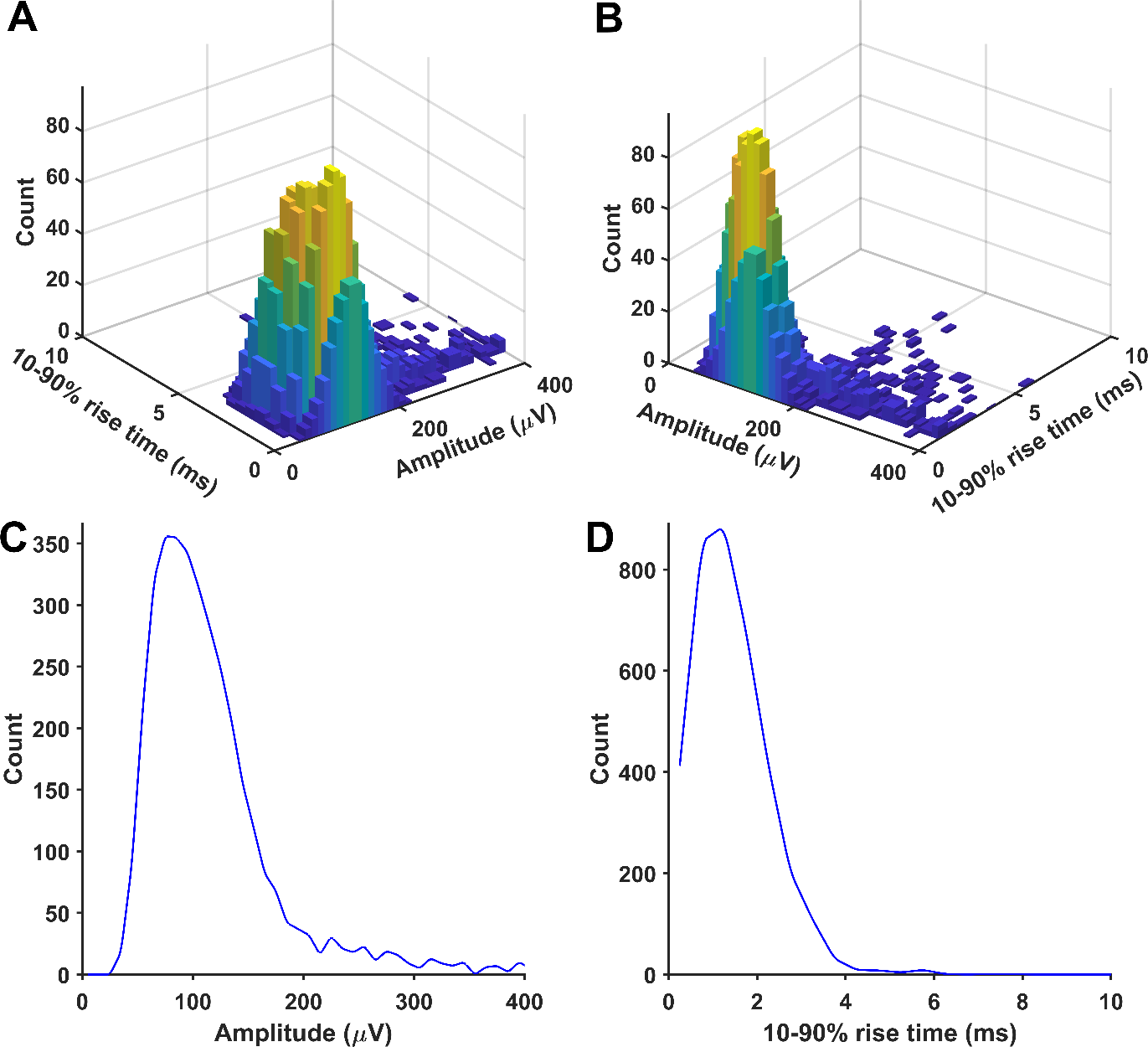
**Supplementary Figure 34:** An example of a distribution of randomly selected simulated minis that was used to produce a close match between distributions of mini-like events detected in a ’noise with simulated minis’ voltage trace (30 µV lower limit on simulated amplitudes) and a ’noise with real minis’ recording for cell p122a (layer 2/3).

Panels as for Supplementary Figure 28.


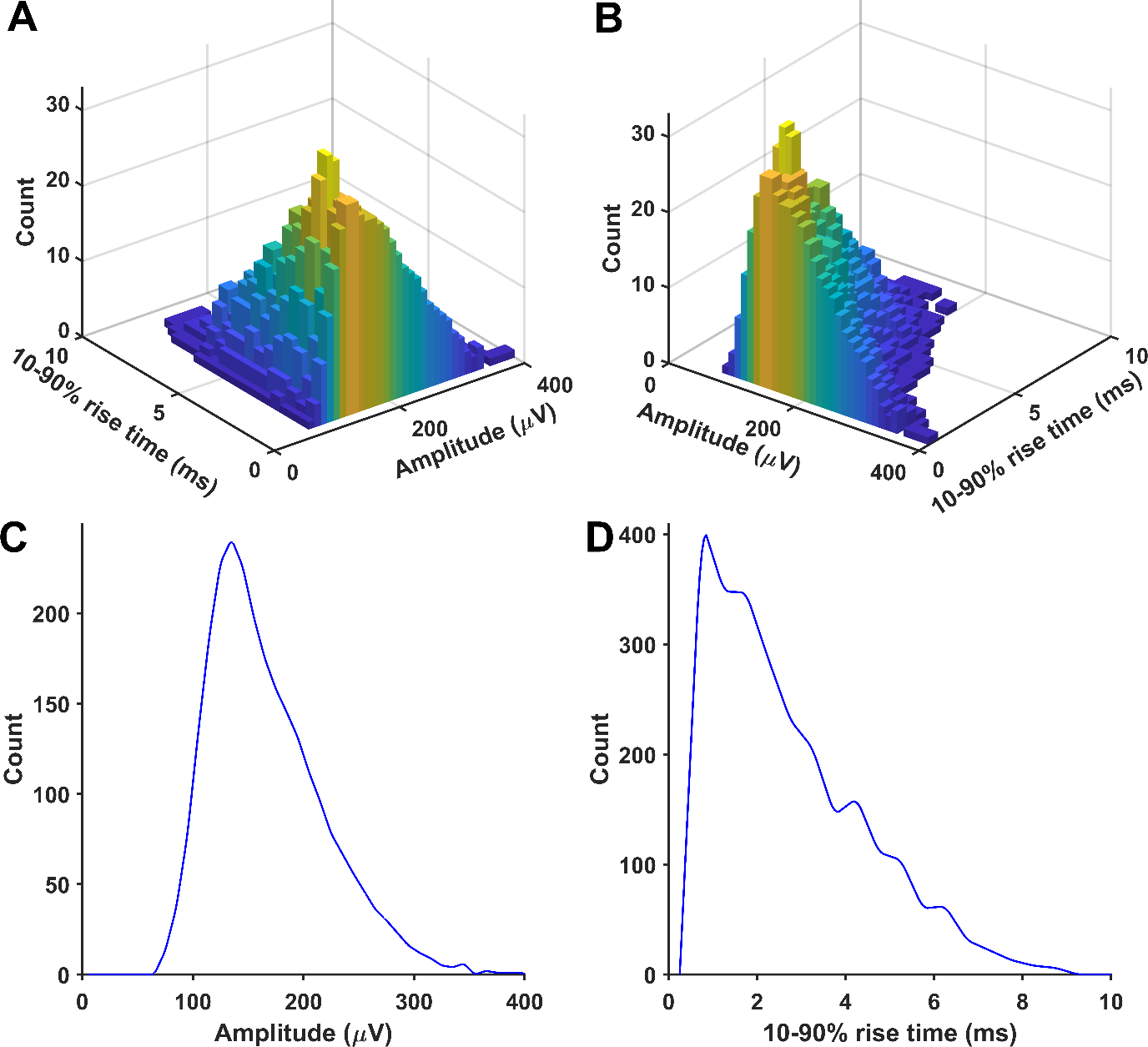
**Supplementary Figure 35:** An example of a distribution of randomly selected simulated minis that was used to produce a close match between distributions of mini-like events detected in a ’noise with simulated minis’ voltage trace (40 µV lower limit on simulated amplitudes) and a ’noise with real minis’ recording for cell p124b (layer 5).

Panels as for Supplementary Figure 28.


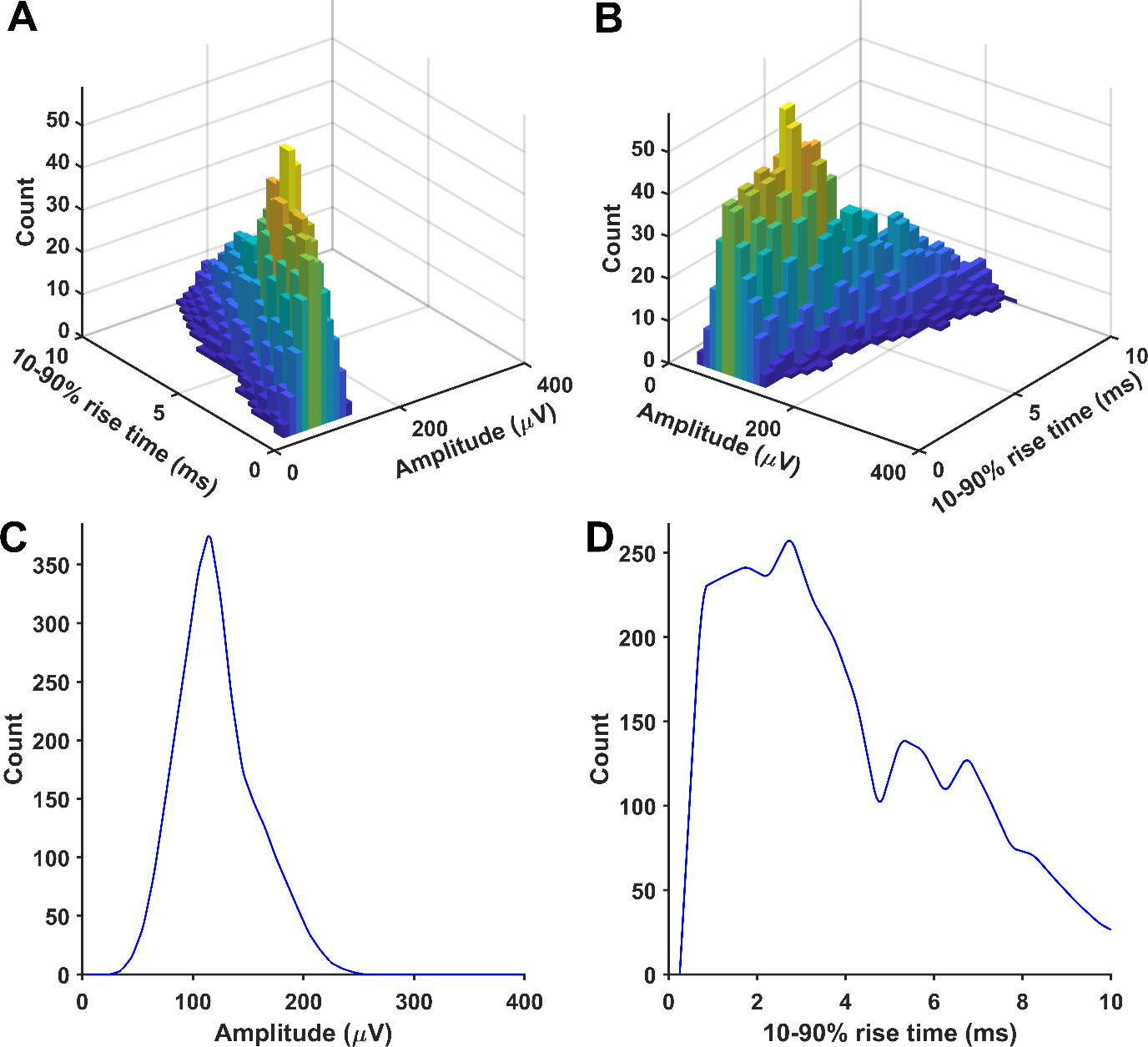
**Supplementary Figure 36:** An example of a distribution of randomly selected simulated minis that was used to produce a close match between distributions of mini-like events detected in a ’noise with simulated minis’ voltage trace (30 µV lower limit on simulated amplitudes) and a ’noise with real minis’ recording for cell p125a (layer 2/3).

Panels as for Supplementary Figure 28.


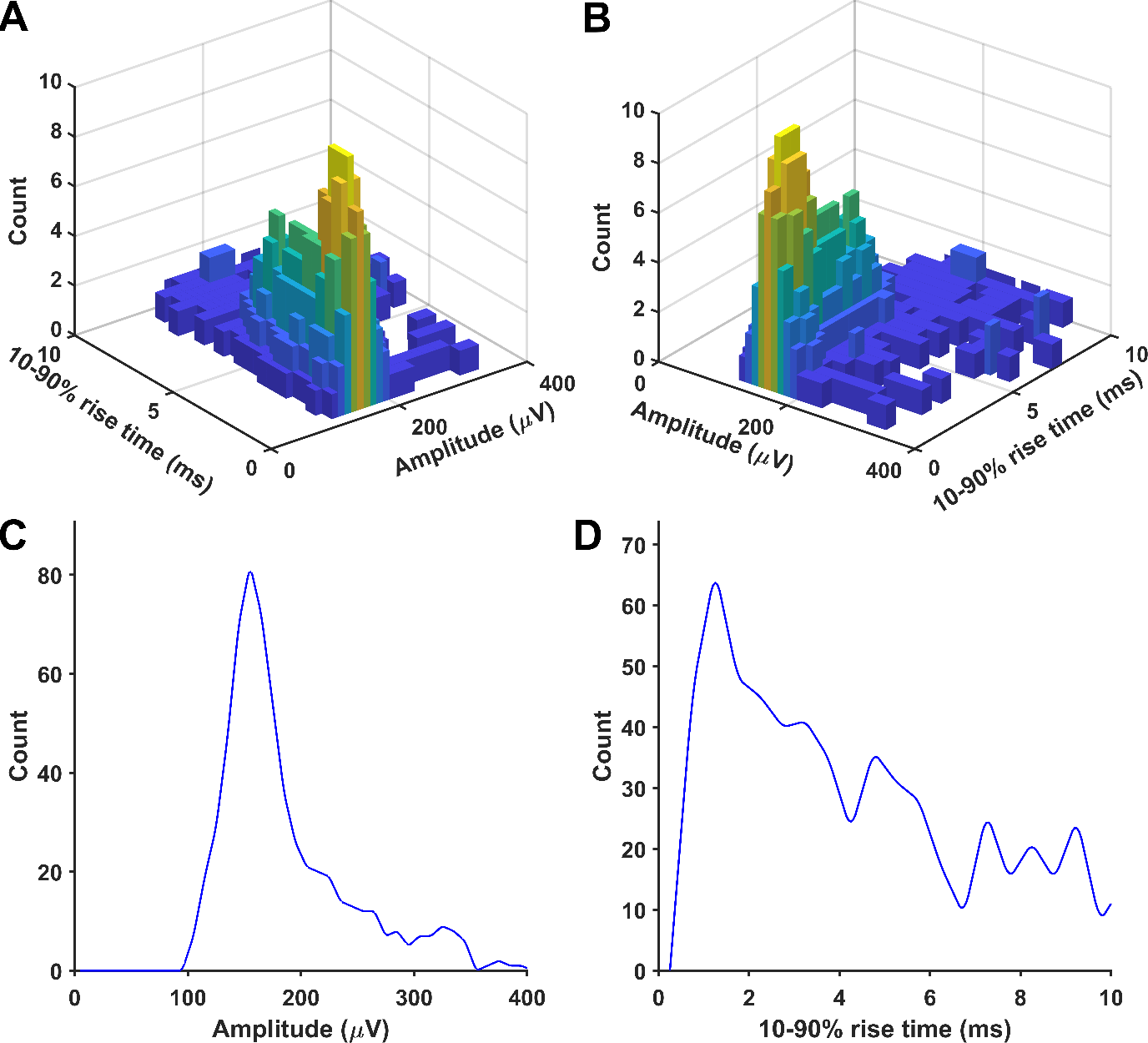
**Supplementary Figure 37:** An example of a distribution of randomly selected simulated minis that was used to produce a close match between distributions of mini-like events detected in a ’noise with simulated minis’ voltage trace (100 µV lower limit on simulated amplitudes) and a ’noise with real minis’ recording for cell p127c (layer 2/3).

Panels as for Supplementary Figure 28.


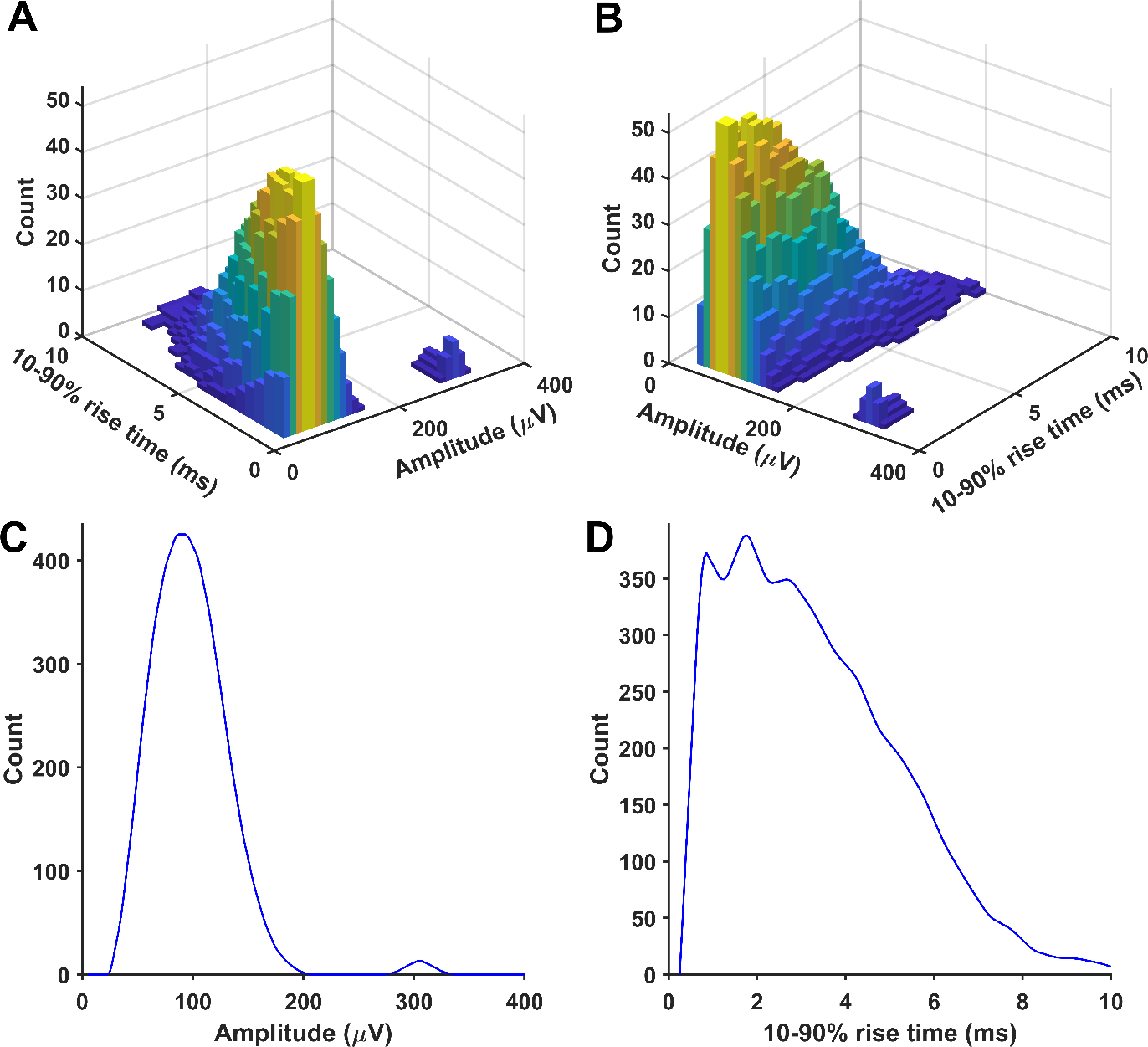
**Supplementary Figure 38:** An example of a distribution of randomly selected simulated minis that was used to produce a close match between distributions of mini-like events detected in a ’noise with simulated minis’ voltage trace (30 µV lower limit on simulated amplitudes) and a ’noise with real minis’ recording for cell p128c (layer 2/3).

Panels as for Supplementary Figure 28.


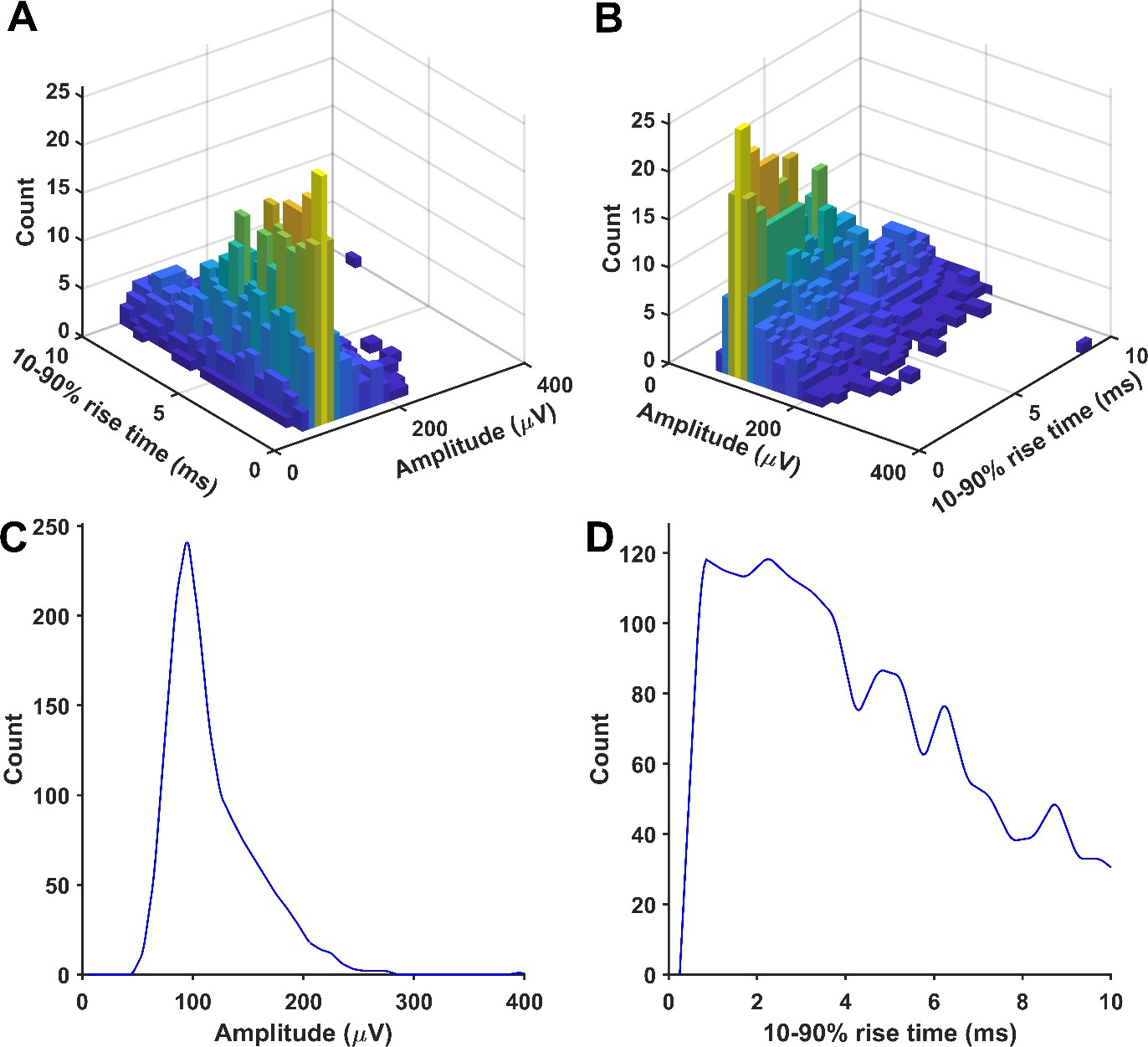
**Supplementary Figure 39:** An example of a distribution of randomly selected simulated minis that was used to produce a close match between distributions of mini-like events detected in a ’noise with simulated minis’ voltage trace (50 µV lower limit on simulated amplitudes) and a ’noise with real minis’ recording for cell p129a (layer 5).

Panels as for Supplementary Figure 28.


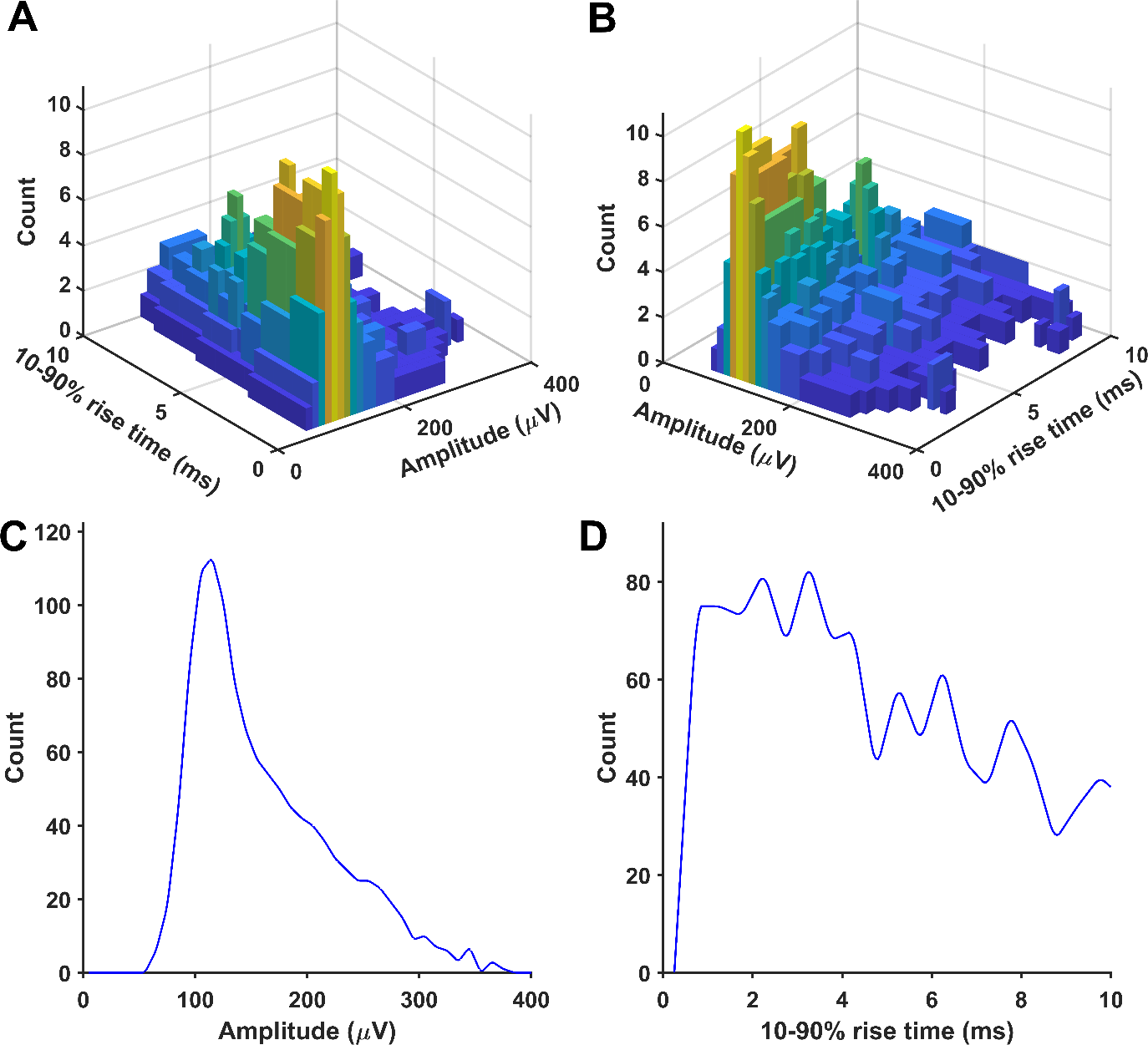
**Supplementary Figure 40:** An example of a distribution of randomly selected simulated minis that was used to produce a close match between distributions of mini-like events detected in a ’noise with simulated minis’ voltage trace (60 µV lower limit on simulated amplitudes) and a ’noise with real minis’ recording for cell p131a (layer 2/3).

Panels as for Supplementary Figure 28.
